# Global, regional, and national burden of ischaemic heart disease from 1990 to 2021: a comprehensive analysis based on the Global Burden of Disease study 2021

**DOI:** 10.7189/jogh.15.04291

**Published:** 2025-12-05

**Authors:** Chenglin Liu, Qifeng Jin, Chaoxin Han, Min Jiao

**Affiliations:** 1 Department of Cardiology, Affiliated Hospital of Jining Medical University, Jining, China; 2 School of Public Health, Jining Medical University, Jining, China

## Abstract

**Background:**

Globally, the issue of ischaemic heart disease (IHD) has emerged as a prominent public health challenge in the ongoing process of ageing. Previous assessments relied upon data constrained by geographical scope and lacking a thorough worldwide evaluation. We aimed to present the incidence, prevalence, death, and disability-adjusted life years (DALYs) due to IHD at global, regional, and national levels from 1990 to 2021, emphasising decomposition and progressive analysis. We aim to provide relevant information to guide health policy decisions, allocate medical resources effectively, and improve patient care protocols for greater efficiency.

**Methods:**

We aimed to accurately depict the health impact of IHD by applying standardised Global Burden of Disease approaches and analysing four key epidemiological indicators: prevalence, incidence, mortality, and DALYs. We quantified temporal trends in the burden of IHD from 1990 to 2021 using the estimated annual percentage change (EAPC) metric. We conducted an in-depth examination of global trends, categorising them by age group, gender, and the sociodemographic index (SDI) to provide a more nuanced understanding. Decomposition analyses of IHD DALYs, which examine the effects of age distribution, population dynamics, and changes in disease patterns, enabled us to accurately quantify the specific contributions of each factor to the overall IHD burden. Using frontier analytical methods, we intended to pinpoint the minimal plausible burden of IHD, contingent on the level of development, as gauged by the SDI.

**Results:**

In 2021, the age-standardised incidence rate (ASIR) of IHD decreased compared with 1990 (EAPC = −0.44; 95% confidence interval = −0.47, −0.42). Moreover, the age-standardised mortality rates (ASMR) and DALYs (ASDR) decreased over time. The overall IHD burden was marginally higher in males than in females. The global rates for prevalence, incidence, deaths, and DALYs related to IHD demonstrated an overall rising trend along with age. Among all regions, the North Africa and Middle East region exhibited the highest ASIR (ASIR = 895.85; 95% uncertainty interval (UI) = 786.65, 1043.49) and age-standardised prevalence rate (ASPR) (ASPR = 6404.84; 95% UI = 5872.02, 7041.08) for IHD in 2021. Central Asia recorded the highest ASMR (ASMR = 265.51; 95% UI = 240.67, 290.42) and ASDR (ASDR = 4864.49; 95% UI = 4415.55, 5338.75) in 2021. Decomposition analysis revealed population growth and ageing as primary factors driving the rise in IHD DALYs. Frontier analysis illuminated ample room for enhancement across the entire development continuum.

**Conclusions:**

The variability in IHD burden is influenced by gender, age, and geographic location. The global burden of IHD has persistently increased during the last three decades, notably among older males. The escalating ageing population and demographic expansion underscore the importance of bolstering public health measures and optimising resource allocation, particularly in etiological investigation, prompt diagnosis, preventive measures, and locally tailored management for IHD.

Globally, approximately one-third of fatalities are attributed to cardiovascular conditions [1], with ischaemic heart disease (IHD) preeminent among these [2]. Additionally known as atherosclerotic cardiovascular disease (CVD) and coronary artery disease, IHD clinically exhibits itself as ischaemic cardiomyopathy and myocardial infarction [3]. The fundamental pathological mechanism of IHD is the development of atherosclerosis, an inflammatory arterial condition characterised by lipid accumulation and metabolic shifts driven by various risk factors [4]. A growing population of IHD patients endure chronic disabilities, impacting their quality of life [5].

Specific investigations have shown that the incidence of IHD can be prevented and potentially eliminated when underlying risk factors are effectively managed and mitigated [6–8]. The escalating global burden of IHD stems primarily from the cumulative influence of social-demographic disparities, limited accessibility to healthcare services, and suboptimal functioning of healthcare systems [2]. While prior studies have mapped IHD trends using Global Burden of Disease (GBD) data [9], these analyses have predominantly focused on descriptive summaries of mortality or incidence at broad regional scales. For instance, the 2017 GBD report on CVDs provided global mortality estimates but lacked a granular decomposition of drivers behind burden shifts [10]. Consequently, critical gaps remain: existing literature rarely quantifies the relative contributions of population ageing *vs.* epidemiological transitions to rising disability-adjusted life years (DALYs), fails to establish benchmarks for achievable burden based on sociodemographic development, and often overlooks cross-indicator disparities (*e.g.* why regions with similar incidence rates exhibit divergent mortality outcomes).

The 2021 iteration of the GBD study offers an updated analytical structure for assessing disease impacts [11]. To address these limitations, we leverage GBD data from 1990 to 2021 to deliver three novel contributions. Unlike prior reports, we synthesise four key epidemiological indicators (*i.e.* prevalence, incidence, mortality, and DALYs) across 204 countries/territories, stratified by age, gender, and sociodemographic index (SDI), to reveal previously underexamined disparities. We conduct a demographic-epidemiological decomposition analysis to isolate the specific contributions of population growth, ageing, and epidemiological shifts to changes in IHD DALYs, providing insights into why burden evolves, beyond descriptive trends. Lastly, we introduce frontier analysis to benchmark IHD burden against SDI, identifying the minimum achievable burden for each development stratum to inform targeted resource allocation.

## METHODS

### Data source

In our examination of the 2021 GBD study, we acquired recurring cross-sectional data via the Global Health Data Exchange [12]. This data encompasses the worldwide impact of 369 diseases and injuries across 204 countries and territories, from 1990 to 2021 [13,14]. From GBD 2021, we derived estimates and their 95% uncertainty intervals (UI) for prevalence, incidence, mortality, DALYs, and age-standardised rates (ASRs) of IHD burden. ASRs were reported per 100 000 population based on the GBD standard.

GBD 2021 computed the SDI for each country, a composite metric reflecting socio-economic factors affecting health outcomes. It is the geometric mean of fertility rate (<25), average years of education (≥15), and lagged per capita income, with 0 indicating low education, income, and high fertility [15]. The SDI is segmented into five tiers: the low, low-middle, middle, high-middle, and high. For our secondary data analysis, no ethical clearance or informed consent from an institutional review board/ethics committee was necessary.

### Decomposition analysis

We conducted a decomposition analysis to quantify the contributions of population dynamics and epidemiological changes to the time trends of IHD DALYs (1990–2021). This analysis employed an additive, incremental decomposition framework to untangle the demographic and epidemiological drivers of disease burden, ensuring that the total change in DALYs equals the sum of the individual component contributions and thereby avoiding ambiguity in interpreting overlapping effects. The decomposition structure was as follows:

△DALYs = △(population size) + △(age structure) + △(epidemiologic changes)

△(population size) represents the change in DALYs attributed to overall population growth, △(age structure) represents changes due to population ageing. and △(epidemiologic changes) represents the residual change after the first two components.

We validated our results through cross-validation by reversing the time direction (2021–1990). The discrepancy between forward and backward estimates was less than 5%, confirming the stability and robustness of our findings. This method ensures the reliability of the decomposition results and accurately reflects the underlying population and epidemiological dynamics.

### Frontier analysis

To explore the efficiency of different countries in managing the burden of IHD, we conducted a frontier analysis. This involved constructing an efficient frontier using Stochastic frontier analysis to identify countries with the best performance relative to their SDI levels. The assumptions underlying this model include that SDI fully reflects the developmental factors influencing the burden of IHD and that SDI is significantly correlated with healthcare expenditure.

### Statistical analysis

We examined the standardised incidence, prevalence, mortality, and DALYs associated with IHD, using the estimated annual percentage change (EAPC) as a metric to quantify trends in the burden of IHD in 1990–2021. We constructed a linear regression model to examine the correlation between temporal progression and the logarithm of the age-adjusted rate, formulated as *y = α + βx + ε*, where α and β are coefficients, calendar year is denoted by *x*, and ε represents the residual error. We computed EAPC as 100 × [exp(β) − 1], with a 95% confidence interval (CI) derived from the regression analysis [16]. An increasing ASR trend emerged when both EAPC’s boundaries were positive, whereas a declining trend was observed when both were negative. In other scenarios, the ASR was deemed stable [17]. To account for the uncertainty in the GBD 2021 estimates and propagate this variability into the EAPC, decomposition, and frontier analysis, we implemented a Monte Carlo simulation. This framework uses the 95% UI from GBD 2021 for IHD rates by age group, treating each rate as a random variable sampled from a log-normal distribution. For preprocessing the data, we employed the *R* libraries ‘tidyverse’ and ‘stringr’ for cleaning, and subsequently used the ‘ggplot2’ *R* package for visualising the data. To address the risk of false positives arising from multiple tests, we set strict significance thresholds in our statistical analysis. The significance level for the set *P*-value was 0.05, and we applied the Bonferroni correction to adjust for multiple tests. Specifically, the significance threshold for each independent test was 0.05 divided by the total number of tests to control the family error rate. We reported the original *P*-value and adjusted *P*-value, and considered the results statistically significant only when the adjusted *P*-value was below the new significance level. We used *R,* version 4.3.0 (R Core Team, Vienna, Austria) for all analyses.

## RESULTS

### Global burden of IHD

In 2021, there were 31 872 778 cases (95% UI = 13 180 529, 18 849 479) of IHD globally, showing a significant increase of 1.02% compared with 1990. The age-standardised incidence rate (ASIR) of IHD was 372.90 per 100 000 (95% UI = 307.95, 444.19). Moreover, ASIR decreased from 1990 to 2021 (EAPC = −0.44; 95% CI = −0.47, −0.42), resulting in an overall 11.12% reduction. However, the global prevalence of IHD was 254 276 268 cases (95% UI = 221 446 458, 295 493 093), with age-standardised prevalence rates (ASPRs) of 2946.38 per 100 000 (95% UI = 2572.69, 3424.32). Between 1990 and 2021, the ASPR rose by 1.43% (95% UI = −2.55, 5.94). Surprisingly, there was no change in the EAPC value (EAPC = 0; 95% CI = −0.02, 0.03). The count of deaths stood at 8 991 637 (95% UI = 8 264 123, 9 531 130), with an age-standardised mortality rate (ASMR) of 108.73 per 100 000 individuals (95% UI = 99.6, 115.38), demonstrating a decrease of 31.57% (EAPC = −1.30%; 95% CI = −1.34, −1.26) from 1990 to 2021. The age-standardised DALY rate (ASDR) for IHD remained at 2212.16 per 100 000 (95% UI = 2075.54, 2327.61), signifying a 28.81% reduction (EAPC = −1.20%; 95% CI = −1.25, −1.16) since 1990 (**Table 1**).

**Table 1:** Age-standardised prevalence, incidence, deaths, and DALYs of IHD in 2021, with percentage changes and EAPC (1990–2021)

	Incidence			Prevalence			Death			DALY		
	**ASR (95% UI)**	**% changes (95% CI)**	**EAPC (95% CI)**	**ASR (95% UI)**	**% changes (95% CI)**	**EAPC (95% CI)**	**ASR (95% UI)**	**% changes (95% CI)**	**EAPC (95% CI)**	**ASR (95% UI)**	**% changes (95% CI)**	**EAPC (95% CI)**
**Global**	372.90 (307.95, 444.19)	−11.12 (−12.69, −9.54)	−0.44 (−0.47, −0.42)	2946.38 (2572.69, 3424.32)	1.43 (−2.55, 5.94)	0 (−0.02, 0.03)	108.73 (99.6, 115.38)	−31.57 (−34.86, −28.33)	−1.3 (−1.34, 1.26)	2212.16 (2075.54, 2327.61)	−28.81 (−32.55, −25.16)	−1.20 (−1.25, −1.16)
**Low SDI**	444.61 (362.90, 537.75)	−5.68 (−7.35, −4.11)	−0.3 (−0.36, −0.25)	3162.53 (2763.3, 3679.6)	2.01 (−1.84, 6.06)	0.03 (0.02, 0.04)	116.41 (105.21, 127.69)	−2.59 (−11.63, 7.41)	0.01 (−0.10, 0.11)	2464.12 (2235.87, 2725.2)	−7.67 (−16.52, 1.58)	−0.26 (−0.34, −0.18)
**Low-middle SDI**	515.60 (433.64, 614.95)	−3.00 (−4.57, −1.20)	−0.09 (−0.13, −0.05)	3941.42 (3448.94, 4577.03)	6.03 (2.32, 10.24)	0.22 (0.20, 0.23)	142.1 (131.3, 151.87)	0.79 (−7.41, 10.02)	0.16 (0.09, 0.23)	3138.58 (2912.65, 3360.62)	−2.58 (−10.90, 6.63)	0.02 (−0.03, 0.08)
**Middle SDI**	403.84 (330.37, 481.69)	5.50 (4.00, 6.91)	0.22 (0.15, 0.28)	3226.3 (2806.67, 3798.24)	12.48 (8.22, 18.08)	0.39 (0.37, 0.42)	118.71 (107.23, 127.8)	−6.57 (−13.56, 1.09)	−0.12 (−0.19, −0.04)	2351.21 (2174.84, 2514.63)	−9.34 (−16.11, −1.76)	−0.27 (−0.32, −0.23)
**High-middle SDI**	404.44 (331.92, 480.98)	−12.60 (−15.14, −10.16)	−0.58 (−0.69, −0.47)	3217.58 (2814.34, 3742.18)	0.63 (−3.75, 5.56)	−0.06 (−0.09, −0.02)	127.5 (115.03, 137.72)	−34.26 (−38.77, −29.63)	−1.56 (−1.77, −1.36)	2301.49 (2122.97, 2482.46)	−35.89 (−40.49, −30.92)	−1.74 (−2.00, −1.48)
**High SDI**	195.63 (164.52, 231.53)	−43.05 (−45.08, −40.90)	−2.04 (−2.28, −1.81)	1671.6 (1475.88, 1910.43)	−26.42 (−28.61, −23.94)	−1.21 (−1.35, −1.07)	58.45 (52.18, 61.92)	−62.91 (−64.24, −61.95)	−3.43 (−3.53, −3.32)	1134.02 (1053.56, 1186.53)	−61.16 (−62.27, −60.06)	−3.26 (−3.37, −3.14)
**Andean Latin America**	233.09 (188.74, 282.95)	−6.76 (−10.38, −3.26)	−0.31 (−0.38, −0.23)	2236.34 (1992.49, 2499.74)	9.53 (4.21, 14.76)	0.34 (0.29, 0.39)	58.17 (49.56, 69.33)	−37.41 (−46.42, −26.57)	−1.74 (−2.12, −1.36)	1150.61 (976.72, 1369.62)	−38.08 (−47.58, −27.01)	−1.77 (−2.14, −1.39)
**Australasia**	220.63 (181.56, 267.7)	−39.71 (−44.42, −34.46)	−1.7 (−1.98, −1.41)	1966.99 (1749.66, 2225.89)	−19.77 (−25.35, −14.57)	−0.85 (−1.01, −0.69)	46.67 (40.23, 50.21)	−73.60 (−75.33, −72.39)	−4.52 (−4.62, −4.43)	812.69 (739.29, 858.37)	−74.89 (−76.16, −73.82)	−4.68 (−4.82, −4.54)
**Caribbean**	360.97 (298.09, 428.10)	−12.49 (−15.20, −9.62)	−0.48 (−0.59, −0.37)	3184.00 (2861.96, 3542.79)	0.05 (−4.45, 4.30)	−0.04 (−0.10, 0.02)	112.5 (100.51, 126.54)	−40.46 (−46.79, −34.08)	−1.71 (−1.90, −1.51)	2397.8 (2112.34, 2730.37)	−35.60 (−43.19, −27.42)	−1.43 (−1.63, −1.22)
**Central Asia**	801.56 (731.97, 893.80)	24.86 (19.52, 31.26)	0.7 (0.54, 0.86)	4408.38 (4040.59, 4801.11)	7.63 (4.15, 11.31)	0.24 (0.21, 0.27)	265.51 (240.67, 290.42)	−17.15 (−24.51, −10.22)	−1.01 (−1.29, −0.74)	4864.49 (4415.55, 5338.75)	−21.63 (−29.10, −14.32)	−1.31 (−1.65, −0.98)
**Central Europe**	322.44 (280.41, 371.50)	−34.82 (−37.66, −31.89)	−1.82 (−2.03, −1.6)	3192.86 (2824.44, 3571.8)	−15.16 (−17.91, −11.93)	−0.77 (−0.87, −0.66)	139.98 (126.84, 148.91)	−48.64 (−51.64, −45.75)	−2.46 (−2.56, −2.35)	2471.23 (2288.2, 2635.38)	−52.46 (−55.37, −49.49)	−2.75 (−2.86, −2.64)
**Central Latin America**	309.82 (253.98, 372.65)	−9.71 (−11.83, −7.79)	−0.42 (−0.48, −0.36)	2625.17 (2306.27, 2997.16)	−2.57 (−6.18, 1.28)	−0.15 (−0.18, −0.11)	103.69 (92.48, 114.59)	−17.53 (−24.91, −9.28)	−0.76 (−1.00, −0.53)	2016.97 (1821.53, 2238.28)	−17.53 (−25.61, −8.61)	−0.82 (−1.05, −0.59)
**Central Sub-Saharan Africa**	344.02 (286.71, 408.91)	−7.94 (−12.47, −3.75)	−0.39 (−0.43, −0.35)	2151.75 (1934.11, 2416.46)	−5.41 (−10.49, −0.40)	−0.24 (−0.28, −0.2)	119.34 (93.7, 150.29)	−11.54 (−29.08, 9.10)	−0.57 (−0.66, −0.49)	2433.06 (1902.53, 3100.83)	−13.61 (−31.76, 8.31)	−0.64 (−0.73, −0.56)
**East Asia**	363.64 (291.86, 437.51)	14.97 (12.56, 17.28)	0.62 (0.47, 0.77)	3031.24 (2597.74, 3606.16)	19.62 (13.72, 26.68)	0.61 (0.54, 0.69)	108.9 (91.18, 125.79)	15.95 (−2.57, 38.06)	0.90 (0.59, 1.21)	1839.92 (1541.21, 2135.32)	3.90 (−13.94, 26.42)	0.46 (0.22, 0.72)
**Eastern Europe**	714.22 (578.98, 859.74)	0.51 (−2.86, 3.83)	−0.23 (−0.49, 0.03)	4942.65 (4299.11, 5766.77)	9.08 (4.44, 14.27)	0.20 (0.12, 0.29)	252.89 (226.96, 277.15)	−21.75 (−28.06, −15.22)	−1.24 (−1.69, −0.78)	4687.7 (4267.79, 5115.68)	−21.32 (−27.88, −14.63)	−1.34 (−1.86, −0.81)
**Eastern Sub-Saharan Africa**	314.40 (250.55, 386.71)	−2.61 (−5.04, −0.10)	−0.22 (−0.27, −0.16)	2224.88 (1931.93, 2554.41)	5.24 (1.30, 9.56)	0.12 (0.10, 0.14)	72.16 (62.09, 82.99)	3.92 (−12.59, 21.14)	−0.05 (−0.14, 0.03)	1536.64 (1333.12, 1773.89)	−1.27 (−17.53, 16.04)	−0.25 (−0.34, −0.16)
**High-income Asia Pacific**	92.40 (73.46, 115.10)	−13.31 (−16.91, −9.60)	−0.58 (−0.78, −0.38)	821.73 (714.58, 948.26)	−15.10 (−19.36, −10.90)	−0.77 (−0.88, −0.66)	25.56 (21.78, 27.65)	−61.87 (−63.81, −60.42)	−3.04 (−3.20, −2.88)	492.99 (446.93, 520.22)	−57.71 (−59.00, −56.48)	−2.75 (−2.84, −2.67)
**High-income North America**	174.12 (147.07, 203.07)	−61.73 (−65.26, −57.33)	−3.55 (−3.82, −3.27)	1494.62 (1280.2, 1746.9)	−47.57 (−50.00, −44.80)	−2.47 (−2.65, −2.28)	75.85 (67.17, 80.6)	−57.32 (−58.39, −56.41)	−3.09 (−3.24, −2.94)	1461.9 (1350.51, 1526.21)	−56.33 (−57.32, −55.40)	−2.98 (−3.13, −2.84)
**North Africa and the Middle East**	895.85 (786.65, 1043.49)	−8.99 (−11.60, −6.43)	−0.42 (−0.49, −0.34)	6404.84 (5872.02, 7041.08)	−0.47 (−4.24, 3.30)	−0.07 (−0.09, −0.04)	202.85 (180.59, 223.68)	−26.28 (−32.58, −19.34)	−1.03 (−1.07, −0.98)	4023.22 (3581.71, 4507.47)	−30.19 (−37.11, −22.70)	−1.24 (−1.27, −1.20)
**Oceania**	378.32 (297.30, 469.96)	0.77 (−2.82, 4.64)	0.03 (0.02, 0.05)	2912.09 (2623.82, 3229.74)	3.60 (−0.90, 8.28)	0.14 (0.12, 0.16)	170.89 (145.43, 201.15)	−6.39 (−20.19, 9.95)	−0.18 (−0.22, −0.14)	3962.77 (3323.16, 4723.71)	−6.96 (−22.10, 13.49)	−0.19 (−0.24, −0.14)
**South Asia**	580.24 (472.94, 704.59)	−3.33 (−4.84, −1.65)	−0.13 (−0.21, −0.05)	4455.73 (3796.73, 5339.2)	6.77 (2.24, 12.17)	0.22 (0.21, 0.23)	149.14 (136.97, 161.16)	9.35 (−2.23, 23.35)	0.44 (0.30, 0.57)	3351.09 (3075.41, 3616.42)	2.27 (−8.99, 15.14)	0.17 (0.07, 0.26)
**Southeast Asia**	231.58 (193.09, 275.95)	−3.47 (−5.96, −1.03)	−0.05 (−0.12, 0.02)	2088.43 (1847.37, 2376.73)	4.32 (0.74, 7.66)	0.17 (0.14, 0.19)	110.92 (100.18, 120.2)	−3.32 (−14.68, 9.88)	−0.10 (−0.18, −0.03)	2415.55 (2177.87, 2635.3)	−5.24 (−16.54, 6.96)	−0.16 (−0.22, −0.11)
**Southern Latin America**	201.48 (172.90, 236.15)	−36.04 (−39.62, −32.21)	−1.68 (−1.93, −1.43)	1538.85 (1388.1, 1718.57)	−14.79 (−19.15, −10.34)	−0.71 (−0.79, −0.62)	54.41 (50.08, 57.26)	−63.59 (−65.06, −62.29)	−2.92 (−3.08, −2.76)	1070.86 (1012.57, 1113.22)	−62.05 (−63.35, −60.76)	−2.86 (−3.00, −2.72)
**Southern Sub-Saharan Africa**	378.48 (299.20, 467.87)	−6.13 (−8.60, −3.59)	−0.36 (−0.44, −0.29)	2780.69 (2387.17, 3277.9)	−2.73 (−6.67, 1.49)	−0.19 (−0.24, −0.14)	83.44 (76.93, 90.19)	9.85 (0.10, 27.20)	0.27 (−0.13, 0.66)	1689.53 (1565.07, 1832.24)	3.88 (−5.23, 18.38)	0.11 (−0.30,0.52)
**Tropical Latin America**	167.80 (136.41, 200.76)	−14.76 (−17.16, −12.17)	−0.37 (−0.44, −0.29)	1977.99 (1672.38, 2324.96)	1.20 (−2.74, 5.42)	0.07 (0.04, 0.10)	64.49 (58.98, 67.84)	−52.55 (−54.25, −50.79)	−2.27 (−2.37, −2.18)	1476.14 (1385.48, 1533.17)	−49.10 (−50.85, −47.11)	−2.13 (−2.20, −2.06)
**Western Europe**	172.46 (147.80, 203.44)	−43.52 (−47.23, −39.65)	−1.89 (−2.00, −1.79)	1480.14 (1305.92, 1679.62)	−24.01 (−27.63, −19.77)	−1.05 (−1.11, −0.98)	47.27 (41.45, 50.42)	−68.11 (−69.86, −67.08)	−3.89 (−3.99, −3.80)	843.77 (775.27, 886.1)	−69.21 (−70.43, −68.34)	−4.00 (−4.10, −3.90)
**Western Sub-Saharan Africa**	379.25 (303.36, 464.67)	0.84 (−2.26, 3.26)	−0.07 (−0.10, −0.03)	2624.72 (2284.48, 3000.35)	9.40 (5.73, 13.36)	0.32 (0.30, 0.34)	105.97 (92.83, 120.17)	0.64 (−12.77, 19.53)	0.02 (−0.10, 0.15)	2029.03 (1760.44, 2334.06)	−2.56 (−16.26, 16.73)	−0.10 (−0.24, 0.04)

ASR – age-standardised rate, CI – confidence interval, DALY – disability-adjusted life-year, EAPC – estimated annual percentage change, IHD – ischaemic heart disease, UI – uncertainty interval

### Global trends by gender

**Figure 1:**
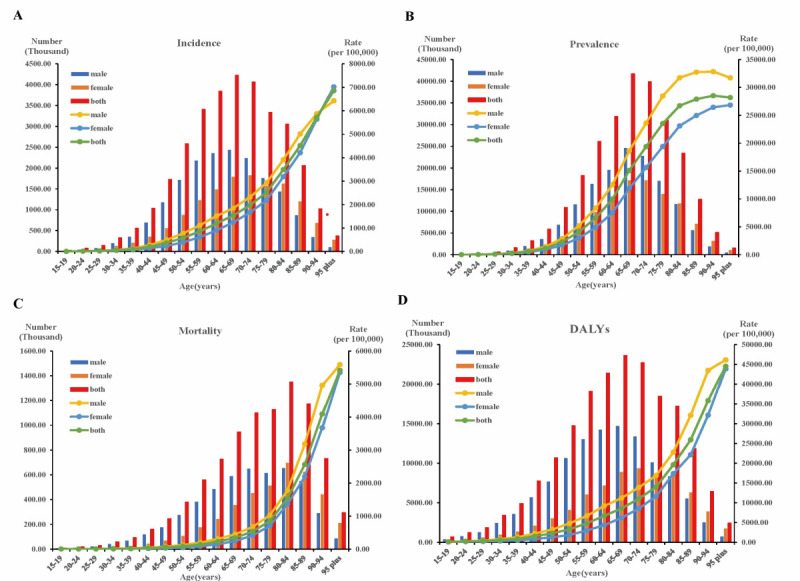
IHD burden by age and gender globally in 2021. **Panel A.** Incidence. **Panel B.** Prevalence. **Panel C.** Deaths. **Panel D.** DALYs. DALY – disability-adjusted life-year, IHD – ischaemic heart disease.

**Figure 2:**
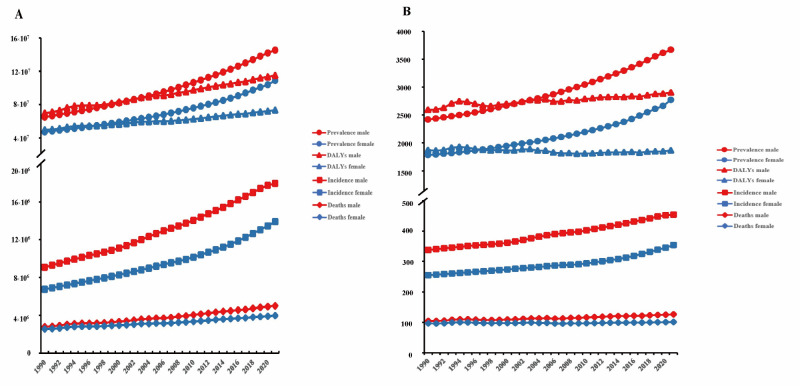
Incidence, prevalence, deaths, and DALYs in males and females,1990–2021. **Panel A.** Global number. **Panel B.** Crude rate. DALY – disability-adjusted life-year.

During 1990–2021, the numbers and approximate ratios of incidence, prevalence, and deaths for both males and females showed an upward trend. Considering the comprehensive amounts and trends, the overall IHD burden was marginally higher in males than in females (**Figure 2**). In 2021, considerable variations in disease burden emerged between males and females among different age groups. Notably, the number of incidences, prevalence, deaths, and DALYs was higher in females than in males after the age of 80 (**Figure 1**). In 2021, the incidence rate of IHD for females was 301.57 per 100 000 (95% UI = 248.57, 360.73), while it was 450.39 per 100 000 (95% UI = 373.66, 534.62) for males (Tables S1 and S2 in the **Online Supplementary Document**). The prevalence rate for females was 2357.61 per 100 000 (95% UI = 2063.31, 2751.95), and for males it was 3610.2 per 100 000 (95% UI = 3153.05, 4164.95). The ASMR was 85.32 per 100 000 (95% UI = 75.90, 92.31) for females and 136.84 per 100 000 (95% UI = 127.37, 145.90) for males. The DALYs rate for females was 1596.14 per 100 000 (95% UI = 1463.98, 1706.95), and it was 2890.65 per 100 000 (95% UI = 2714.81, 3091.32) for males.

### Global trends by age distribution

The global crude rates for prevalence, incidence, deaths, and DALYs related to IHD showed an overall upward trend with age. From the 15–19 age range, the burden imposed by IHD progressively escalated as one advanced in years, particularly among the elderly aged ≥60. The number of sufferers with IHD in each age cohort initially increased and subsequently decreased, reaching a peak at 65–69 years, whereas deaths reached a peak at 80–84 years (**Figure 1**; Table S3 in the **Online Supplementary Document**). The incidence rate increased with age (**Figure 1**, Panel A). Additionally, the rate of mortality and DALYs was prone to increase with age (**Figure 1**, Panels C and D). The incidence rate due to IHD increased significantly with age, peaking at 90–94 years before declining (**Figure 1**, Panel B).

### Regional burden of IHD

**Figure 3:**
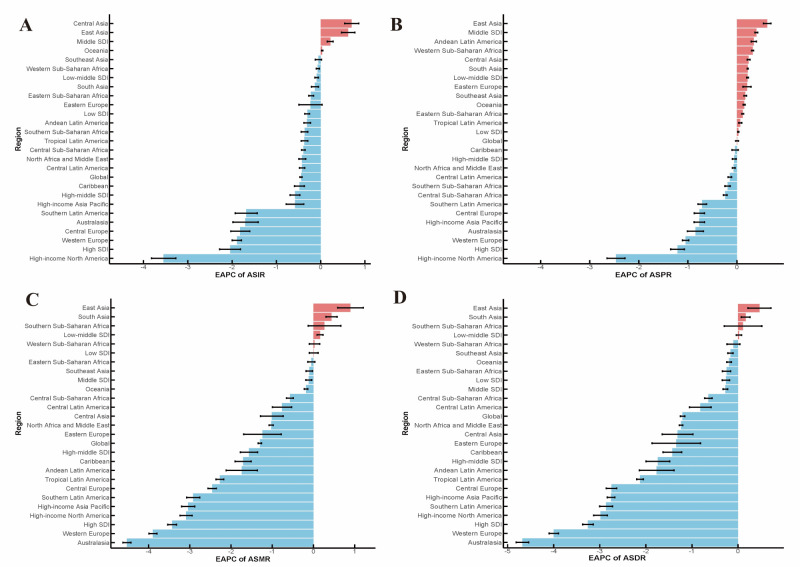
The EAPCs for IHD across 21 regions in 2021. **Panel A.** ASIR. **Panel B.** ASPR. **Panel C.** ASMR. **Panel D.** ASDR. ASDR – age-standardised disability-adjusted life year rate, ASIR – age-standardised incidence rate, ASMR – age-standardised mortality rate, ASPR – age-standardised prevalence rate, EAPC – estimated annual percentage change, IHD – ischaemic heart disease.

Among 27 regions, the North Africa and Middle East region exhibited the greatest ASIR for IHD, amounting to 895.85 per 100 000 (95% UI = 786.65, 1043.49), which was much higher compared to the global average of 372.90 per 100 000 (95% UI = 307.95, 444.19) (**Table 1**). Except for Central Asia, East Asia, Middle SDI, and Oceania, the ASIR in the remaining regions decreased significantly. Markedly, High-income North America saw a considerable reduction in ASIR for IHD during the period from 1990 to 2021, with an EAPC of −3.55 (95% CI = −3.82, −3.27) (**Figure 3**, Panel A).

The highest ASPR for IHD was documented in North Africa and the Middle East (ASPR = 6404.84; 95% UI = 5872.02, 7041.08), followed by Eastern Europe (ASPR = 4942.65; 95% UI = 4299.11, 5766.77), and South Asia (ASPR = 4455.73; 95% UI = 3796.73, 5339.2) (**Table 1**). The most substantial decrease in ASPR of −2.47 (95% UI = −2.65, −2.28) occurred in high-income North America. It is worth noting that, from 1990 to 2021, while ASMR remained unchanged globally, it changed in all other regions. Half of the regions witnessed a downward trend in ASPR, while the other half saw an upward trend (**Figure 3**, Panel B).

The greatest ASMR for IHD was detected in Central Asia (ASMR = 265.51;95% UI = 240.67, 290.42), trailed by Eastern Europe (ASMR = 252.89; 95% UI = 226.96, 277.15), and followed by the North Africa and Middle East (ASMR = 202.85; 95% UI = 180.59, 223.68) (**Table 1**). From 1990 to 2021, except for the unaltered ASMR in low SDI, all the remaining regions exhibited either an upward or a downward trend. The ASMR in regions such as East Asia, South Asia, Southern Sub-Saharan Africa, low-middle SDI, and Western Sub-Saharan Africa showed an increasing trend, while the rest of the regions showed a decreasing trend. Australasia exhibited the most substantial decrease, with an EAPC of −4.52 (95% CI = −4.62, −4.43) (**Figure 3**, Panel C).

Similar to the ASMR, Central Asia exhibited the highest ASDR for IHD (ASDR = 4864.49; 95% UI = 4415.55, 5338.75), followed by Eastern Europe (ASDR = 4687.7; 95% UI = 4267.79, 5115.68), and North Africa and the Middle East (ASDR = 4023.22; 95% UI = 3581.71, 4507.47) (**Table 1**). It should be noted that East Asia, South Asia, Southern Sub-Saharan Africa and Low-middle SDI remained unchanged in terms of ASDR from 1990 to 2021; however, the other regions not falling within this group presented a trend of decline. Importantly, Australasia demonstrated the greatest decrease with an EAPC of −4.68 (95% CI = −4.82, −4.54) (**Figure 1**, Panel D).

### National burden of IHD

In 2021, the top five nations with the highest ASIRs for IHD were Uzbekistan (ASIR = 1206.01; 95% UI = 1125.02, 1305.45), Syrian Arab Republic (ASIR = 1130.3; 95% UI = 993.53, 1305.6), United Arab Emirates (ASIR = 1084.31; 95% UI = 843.39, 1343.89), Egypt (ASIR = 1063.03; 95% UI = 927.44, 1200.31), and Kuwait (ASIR = 1061.15; 95% UI = 836.06, 1330.52) (Figure S1, Panel A and Table S4 in the **Online Supplementary Document**). In contrast, Portugal (ASIR = 72.93; 95% UI = 61.36, 85.88), Japan (ASIR = 90.63; 95% UI = 71.46, 113.97), Republic of Korea (ASIR = 96.28; 95% UI = 79.26, 116.32), Chile (ASIR = 108.52; 95% UI = 97.55, 128.50), and Brunei Darussalam (ASIR = 115.47; 95% UI = 90.12, 146.75) demonstrated the lowest ASIR (Figure S1, Panel A and Table S4 in the **Online Supplementary Document**). From 1990 to 2021, Uzbekistan (EAPC = 2.52; 95% CI = 2.15, 2.90), Tajikistan (EAPC = 1.09; 95% CI = 1.01, 1.18), and Azerbaijan (EAPC = 1.02; 95% CI = 0.88, 1.17) underwent the most significant rise in the ASIR (Table S4 in the **Online Supplementary Document**). Conversely, the USA (EAPC = −3.66; 95% CI = −3.94, −3.38), Portugal (EAPC = −3.44; 95% CI = −3.78, −3.11), and Poland (EAPC = −2.98; 95% CI = −3.26, −2.69) witnessed the most significant reduction in AISR of IHD.

Kuwait (ASPR = 7806.45; 95% UI = 7141.59, 8556.31), United Arab Emirates (ASPR = 7608.89; 95% UI = 6995.21, 8286.01), Saudi Arabia (ASPR = 7341.64; 95% UI = 6747.53, 7966.8), Iraq (ASPR = 7185.54; 95% UI = 6654.59, 7885.02), and Qatar (ASPR = 7143.84; 95% UI = 6501.02, 7864.41) reported the highest ASPR in 2021 (Figure S1, Panel B and Table S4 in the **Online Supplementary Document**). Relatively similar to the ASIR, Japan (ASPR = 802.34; 95% UI = 686.82, 938.16), Republic of Korea (ASPR = 841.83; 95% UI = 748.12,948.82), Brunei Darussalam (ASPR = 937.69; 95% UI = 832.55, 1056.19), Portugal (ASPR = 947.10; 95% UI = 806.24, 1099.61), and Denmark (ASPR = 1085.82; 95% UI = 948.09, 1240.27) presented the lowest ASPR (Figure S1, Panel B and Table S4 in the **Online Supplementary Document**). Uzbekistan (EAPC = 1; 95% CI = 0.89, 1.11), Equatorial Guinea (EAPC = 0.67; 95% CI = 0.60, 0.74), and the United Republic of Tanzania (EAPC = 0.67; 95% CI = 0.61, 0.73) experienced the largest increase in ASPR for IHD (Table S4 in the **Online Supplementary Document**). On the contrary, the USA (EAPC = −2.57; 95% CI = −2.75, −2.38), New Zealand (EAPC = −1.49; 95% CI = −1.61, −1.37), and Finland (EAPC = −1.49; 95% CI = −1.81, −1.16) witnessed the greatest decrease in ASPR.

The highest ASMR were recorded in Nauru (ASMR = 432.64; 95% UI = 361.02, 517.42), Ukraine (ASMR = 373.47; 95% UI = 291.58, 459.07), Syrian Arab Republic (ASMR = 353.01; 95% UI = 281.64, 432.02), Egypt (ASMR = 347.73; 95% UI = 297.31, 402.14), Turkmenistan (ASMR = 343.68; 95% UI = 280.94, 420). However, the lowest ASMR were documented in San Marino (ASMR = 23.19; 95% UI = 15.63, 32), Japan (ASMR = 25.36; 95% UI = 21.7, 27.29), Republic of Korea (ASMR = 28.27; 95% UI = 22.87, 32.57), France (ASMR = 29.87; 95% UI = 25.89, 32.3), and Taiwan (Province of China) (ASMR = 33.61; 95% UI = 30, 36.21) (Figure S1, Panel C and Table S4 in the **Online Supplementary Document**). Nauru (ASDR = 10681.95; 95% UI = 8619.33, 13238.75), Vanuatu (ASDR = 7189.7; 95% UI = 6037.23, 8411.26), Egypt (ASDR = 6924.84; 95% UI = 5844.94, 8119.21), Syrian Arab Republic (ASDR = 6688.8; 95% UI = 5230.44, 8518.87), and Ukraine (ASDR = 6522.69; 95% UI = 5003.4, 8125.67) possessed the highest ASDR values. Conversely, San Marino (ASDR = 429.12; 95% UI = 298.92, 591.84), Republic of Korea (ASDR = 470.99; 95% UI = 402.59, 526.79), Japan (ASDR = 502.25; 95% UI = 460.02, 526.29), France (ASDR = 555.98; 95% UI = 503.45, 597.26), and Israel (ASDR = 599.57; 95% UI = 537.77, 637.8) had the minimum ASDR (Figure S1, Panel D; Table S4 in the **Online Supplementary Document**). The greatest increases in ASMR and ASDR occurred in Lesotho, Zimbabwe, Kenya, Mozambique, and Honduras (Table S4 in the **Online Supplementary Document**). The greatest decreases in ASMR and ASDR occurred in Denmark, Israel, Norway, the Netherlands, and Estonia.

### Decomposition analysis of IHD epidemiology: ageing, population growth, and epidemiologic changes

The decomposition analysis revealed the relative impact of ageing, population growth, and demographically adjusted epidemiological shifts on IHD DALYs, both globally and across the five SDI regions and 21 GBD regions. Between 1990 and 2021, High-income North America and Western Europe, which have witnessed a decline in demographically adjusted epidemiological change, reduced the burden of deaths and DALYs associated with IHD (Figure S2 in the **Online Supplementary Document**). In contrast, Southeast Asia possessed the highest IHD DALYs, predominantly driven by population growth, followed by ageing (Figure S2 in the **Online Supplementary Document**). In general, a substantial rise in IHD DALYs was observed globally and across the high-middle, middle, low-middle, and low SDI quintiles. However, the most notable increase was observed in the middle and Low-middle SDI quintiles, which showed the greatest increase in overall DALYs over the past 32 years (**Table 2**; Figure S2 in the **Online Supplementary Document**). On a global scale, population growth (109.65%) and ageing (67.14%) contributed to the rise in IHD DALYs from 1990 to 2021. The impact of ageing on total DALY rates was most evident in the High-middle SDI quintile, where it accounted for 154.37%, and diminished to 54.45% in the middle SDI and 23.16% in the low-middle SDI category. Most of the increase in IHD-related DALYs can be attributed to population expansion in Low SDI regions (123.1%), whereas in high SDI countries, the contribution of population growth was notably negative, reaching −102.47%. The global trends in epidemiological shifts, indicative of alterations in age- and population-adjusted mortality and morbidity rates due to IHD, have experienced a notable decline (−76.79%). This reduction was most pronounced within the high-middle SDI quintile (−166.05%), whereas an increase was exclusively observed in the high SDI quintile (338.54%) (**Table 2**; Figure S2 in the **Online Supplementary Document**).

**Table 2:** DALY changes by population-level determinants, 1990–2021

		Change due to population-level determinants (contribution % to the total change), n (%)		
	**Overall difference**	**Ageing**	**Population**	**Epidemiological change**
**Global**	69 197 600.02	46 456 256.22 (67.14)	75 876 337.76 (109.65)	−53 134 993.95 (−76.79)
**High SDI**	−8 459 030.72	11 509 583.23 (−136.06)	8 668 370.79 (−102.47)	−28 636 984.74 (338.54)
**High-middle SDI**	10 973 530.73	16 939 967.99 (154.37)	12 255 524.27 (111.68)	−18 221 961.53 (−166.05)
**Middle SDI**	34 715 722.42	18 904 329.39 (54.45)	20 644 159.59 (59.47)	−4 832 766.56 (−13.92)
**Low-middle SDI**	25 385 595.31	5 878 520.55 (23.16)	20 974 860.03 (82.63)	−1 467 785.28 (−5.78)
**Low SDI**	6 583 352.04	−439 713.61 (−6.68)	8 104 134.32 (123.1)	−1 081 068.67 (−16.42)

DALY – disability-adjusted life-year, SDI – sociodemographic index

### Frontier analysis on IHD DALYs

To obtain a deeper understanding of the potential enhancement in IHD DALY rates attainable based on a country's developmental level, we conducted a frontier analysis using age-standardised DALY rates and SDI, spanning 1990 to 2021 (Figure S3, Panel A in the **Online Supplementary Document**). The boundary line indicates the countries and territories with the most favourable DALY profiles relative to their SDI status. The deviation from this boundary, referred to as the efficiency gap, represents the difference between a country's current DALY status and its potential optimal status; this gap can potentially be reduced or eliminated through the strategic allocation of sociodemographic resources (Figure S3, Panels A and B in the **Online Supplementary Document**). The gap between each country and territory's performance and the efficient frontier was calculated using the 2021 DALYs and SDI. The calculation of the significant disparity from the forefront across all nations and regions was performed using DALY and SDI data from 2021 (Figure S3, Panel and Table S5 in the **Online Supplementary Document**). Generally, as the SDI escalates, the notable discrepancy observed for a particular SDI instance tends to diminish, showcasing reduced dispersion. Among the nations demonstrating the most substantial effective disparity from the benchmark frontier, spanning a range from 9946.83 to 5498.03, the leading ten comprise Nauru, Vanuatu, Egypt, the Syrian Arab Republic, Ukraine, the Marshall Islands, Turkmenistan, the Federated States of Micronesia, Belarus, and the Solomon Islands. This list highlights countries that exhibit notable variation in their performance metrics relative to an established frontier standard. These nations exhibit notably higher IHD DALY rates than others with similar sociodemographic backgrounds. Given their position within the development spectrum, the top ten nations with the lowest DALY rates include France, Israel, Japan, Portugal, San Marino, Spain, Korea, Somalia, Italy, and Niger, thereby exhibiting the smallest effective differences. Notably, the effective differences for the first five countries are zero, whereas those for the remaining five range from 0.27 to 92.74 (Figure S3, Panel B and Table S5 in the **Online Supplementary Document**).

## DISCUSSION

We conducted an exhaustive evaluation of the global IHD burden from 1990 to 2021, uncovering pivotal epidemiological patterns and geographical variations that are of paramount significance for guiding the formulation of public health strategies and the equitable distribution of resources. From 1990 to 2021, the latest information regarding the occurrence, prevalence, mortality, and DALYs associated with IHD was offered at global, regional, and national scales. Additionally, a thorough assessment was implemented, incorporating trend analysis, decomposition methods, and frontier analysis. IHD stands as a major worldwide health concern, contributing significantly to both mortality and disability rates [18]. Previous research has documented the burden of IHD in specific regions [19–21], yet this investigation offers the most recent data spanning 1990 to 2021, encompassing global, regional, and national perspectives on IHD incidence, prevalence, mortality, and DALYs. It further delves into a thorough assessment through trend, decomposition, and frontier analyses, providing a comprehensive understanding of the evolving burden of IHD worldwide.

The incidence of IHD has markedly escalated from 1990 to 2021, making up a large part of CVD [22], which now stands as the third most prevalent cause of mortality worldwide [23]. During this timeframe, the global ASIR for IHD declined. The observed alteration in the worldwide distribution of IHD is likely indicative of population expansion, a shift towards an older demographic profile, and lifestyle changes [6,22]. Furthermore, both ASDR and ASMR related to IHD demonstrated a gradual decline over time. The potential underlying cause for these changes could stem from advancements in medicinal treatment [24], notably the refinement of interventional therapies for CVD, which have seen marked improvements since the 1990s [25]. Early detection and intervention of IHD can effectively reduce the burden of DALYs and mortality associated with IHD. Prompt identification and timely management of IHD can substantially alleviate the disease burden in terms of mortality and related DALYs. To mitigate death and disability associated with IHD, diverse approaches have emerged for identifying high-risk individuals, encompassing identifying biomarkers through laboratory tests [26], imaging the heart via radiography [27], gathering data through questionnaires and interviews [28], employing risk evaluation models [29], and leveraging genomic predictions to foresee potential threats [30].

This investigation revealed declines in the ASIR across 18 distinct regions, while the ASPR declined in 11 regions. Furthermore, ASMR decreased in 17 areas, and DALYs decreased in 18 regions, highlighting a broad improvement in health outcomes. Consistent with a previous study, all 21 regions demonstrated a downward trend in the ASIR for IHD. It is worth noting that the ASIR, ASPR, ASMR, and ASDR of IHD significantly decreased in both the high-SDI and high-middle-SDI regions. However, the ASIR, ASPR, ASMR, and ASDR in low-income GBD regions, such as East Asia and Sub-Saharan Africa, have increased, which is consistent with previous research findings [31–34]. In affluent nations, elevated blood pressure, high lipid levels, diabetes mellitus, and overweight conditions have steadily escalated, posing as significant contributors to a substantial share of mortality rates. These health issues have emerged as primary risk factors, compelling attention to their growing impact [35–38]. Advancements in disease prevention strategies, and the enhanced integration of healthcare systems have played pivotal roles in significantly diminishing the mortality rate associated with IHD within high-income nations. In less economically prosperous nations, the adoption of Western lifestyles accompanied by heightened metabolic vulnerabilities and profound air pollution is prompting an escalation of IHD as a formidable health threat [39–41]. Given the diverse SDI levels, especially in lower-income nations facing complex challenges, tailored prevention and intervention plans grounded in local health resources are imperative.

It is worth noting that the regional differences in trends reflect the impact of specific environmental policies and social changes. For example, in Australasia, the ASMR decreased by 4.52%, benefiting from public health initiatives: stringent tobacco control policies (such as federal smoking bans and excise taxes) significantly reduced smoking rates [42], while the widespread use of statins and vascular reconstruction surgeries has improved the management of risk factors [43]. These measures are consistent with research findings linking healthcare systems and preventive policy frameworks to reduced IHD mortality rates in the region.

In line with prior research, the current investigation found a correlation between advancing age and elevated IHD incidence [44]. The vulnerability observed in older patients may stem from their weakened physical condition, compounded by the coexistence of multiple chronic health conditions, which contribute to their heightened susceptibility. It is worth noting that our stratified analysis shows that among adults aged 80 and older, the female ASPR occasionally exceeds that of males, and this reversal may be due to various factors. First, women typically show a longer life expectancy [45], leading to more elderly women facing the risk of IHD, while the higher mortality rate in elderly men may reduce the number of men available for prevalence estimates [46]. Second, compared to the typical chest pain experienced by men, women more frequently present with atypical symptoms (such as fatigue and jaw pain), which may lead to underdiagnosis in women, and the accumulated low reporting obscures the true prevalence [47]. Lastly, postmenopausal women may exhibit higher healthcare engagement due to age-related comorbidities, which could improve the diagnosis of IHD in the elderly population [48].

Moreover, elderly individuals encountering illness face an intensified load due to their diminishing recuperative abilities [49]. The general worldwide figures for ASIR, ASPR, ASMR, and ASDR tend towards being higher among males in comparison to females, which the finding is consistent with previous studies [50] This observation may be significantly correlated with the fact that globally, men are exposed to higher risks of smoking, high-sodium diets, and particulate matter pollution than women [33]. Additionally, hormonal differences, such as the cardioprotective effects of estrogen in premenopausal women, may contribute to lowering the baseline risk for women [51]. The above results indicate the potential need for specialised preventive initiatives tailored for men and senior citizens.

The burden of IHD varied by degree of development and geographical location. DALYs serve as a superior metric for quantifying the burden of disease, surpassing both prevalence and mortality rates in accuracy [6]. Therefore, we conducted a decomposition analysis and a frontier analysis of IHD using DALY. This analysis of IHD DALYs indicated that the primary contributors to the rise were population expansion and the ageing process, with a moderation, albeit insignificant, stemming from the decline in standardised mortality and morbidity rates across the majority of GBD regions. Given the escalating trend is partly attributed to population growth and the ageing process, where the occurrence and prevalence of IHD increase with advancing age. Consequently, utilising ASRs offers a more revealing insight into the fundamental trends [52]. Based on a prior assessment, the global burden of DALYs surged by 32.4% due to the ageing population and was further augmented by 22.1% due to population growth [22].

The comprehensive analysis revealed three prominent and noteworthy themes. Initially, pressures stemming from demographic expansion, including population growth and ageing, have significantly shifted the burden of IHD across all SDI quintiles. Second, the shift in underlying epidemiological trends has indeed alleviated the burden of IHD to a certain extent. However, this mitigating effect is still insufficient to fully offset the profound impacts of rapid population growth and ageing in most cases. Third, the frontier analysis results of this paper indicated that, even in countries with lower levels of development, several countries demonstrate leading performance in terms of DALYs related to IHD. It showed that a country's position on the development spectrum should not prevent it from adjusting policies and utilising available resources to reduce the burden of IHD.

In our investigation, we also observed that the burden of IHD disproportionately affects nations with less advanced economies [53], and a correlation emerged between age-standardised DALY rates and indicators of healthcare accessibility. Certain scholars propose minimising socioeconomic inequalities as a potential strategy to mitigate the overall burden of illness [54]. The frontier analysis presented a perspective, highlighting that across various stages of development, several countries exhibit a notable gap in IHD-related DALYs compared to the optimal frontier, indicating significant potential for improvement and untapped opportunities to bridge this health disparity. Despite the prevalence of frontier countries across various SDI tiers, those with lower SDI levels stand out for their remarkable achievements, demonstrating exceptional performance amidst limited resources. These countries can potentially serve as models for enhancing health outcomes in resource-constrained environments. Given the contrast, a few countries with high SDI have experienced subpar results (including Germany, Finland, and the USA), suggesting that other dynamics might overshadow the health advancements associated with sociodemographic wealth. Future endeavours ought to be undertaken to identify the pivotal factors driving success in standout countries and the impediments hindering advancement in underperforming countries. Addressing this research deficit could significantly inform initiatives aimed at mitigating the burden of IHD [55].

An exhaustive investigation is undertaken in the present research, providing a holistic appraisal of the most recent global and regional health burdens tied to IHD. This endeavour encompasses a thorough scrutiny of data and trends, aiming to illuminate the intricate interplay between IHD and its various socioeconomic, epidemiological, and geographical dimensions. By synthesising the latest findings and insights, the study endeavours to offer a comprehensive view of the current state of IHD-related burdens, thereby facilitating informed decision-making and targeted interventions aimed at alleviating their impact. Previous literature has merely depicted the disease burden of IHD from a single dimension, such as focusing on adolescents [51,56] or a specific region [57–59]. Drawing on the most recent epidemiological data spanning 31 years from 1990 to 2021, encompassing 204 nations and territories worldwide, this investigation unveils patterns of health burden variation, segmented by geographical region, SDI, gender, and age cohorts.

Despite the insights gained, the present investigation is constrained. Initially, the projections are contingent on available information resources, with the precision of GBD metrics hinging on the quality of pre-existing data within individual nations. The ability to report and forecast IHD statistics across 204 countries might prove insufficient, thereby increasing the likelihood of inaccuracies. Second, our decomposition analyses focused on pivotal epidemiological shifts, the impact of ageing, and population expansion as primary considerations; while acknowledging the presence of other potential influences, they are not addressed within the confines of this study. Third, we should embark on an intensive excavation and research into the high-risk factors contributing to the onset of IHD. By identifying and understanding these factors, we can develop more targeted prevention strategies, early diagnostic tools, and effective treatments.

### Limitations

While the GBD study provides one of the most comprehensive data sets available, it is important to acknowledge the limitations inherent in these data sources. First, there is significant variability in data sparsity across different regions. In low-income countries and areas, data on IHD incidence and mortality rates are often limited, which may introduce uncertainty into estimates for regions with sparse monitoring systems. Second, our analysis did not account for key risk factors such as diet, air pollution, and statin use, which limits our understanding of the details. Third, changes in the diagnostic criteria for IHD over time may also introduce bias. Finally, our reliance on country-level aggregated data may introduce regional bias.

## CONCLUSIONS

As a paramount concern in public health, the prevalence, incidence, mortality, and DALYs of IHD showed substantial cross-country variation, yet the global burden of IHD rose notably from 1990 to 2021. The primary factors driving variations in IHD burden were population growth and ageing, which could exacerbate the projected surge in IHD cases. There existed a pronounced disparity in epidemiological and demographic patterns across geographical regions, with a disproportionate burden of IHD falling on less developed economies characterised by suboptimal health care systems. Moreover, there was a clear link between health issues stemming from IHD and sociodemographic indicators, with resource-constrained nations pioneering the reduction of IHD-related DALYs. This served as an exemplary model for others to alleviate the burden of IHD. The increasing burden of IHD should be reflected in both global and domestic health priorities.

## Additional material


Online Supplementary Document
