# Supplementary material for: Global, regional, and national burden of ischaemic heart disease from 1990 to 2021: a comprehensive analysis based on the Global Burden of Disease study 2021
Source: J Glob Health. 2025 Dec 5;15:04291. doi: 10.7189/jogh.15.04291 (PMC12677242; doi:10.7189/jogh.15.04291)
Supplement: Online Supplementary Document [file jogh-15-04291-s001.pdf]

Supplement to: Liu C, Jin Q, Han C, Jiao M. Global, regional, and national burden of ischaemic heart disease from 1990 to 2021: a comprehensive analysis based on the Global Burden of Disease study 2021. J Glob Health. 2025;15:04291.

Table S1 The number of prevalence, incidence, deaths, and DALYs and corresponding age-standardized rates in male IHD patients worldwide, from 1990 to 2021.

Year	Incidence		Prevalence		Deaths		DALYs	
	Number	Rate	Number	Rate	Number	Rate	Number	Rate
**1990**	9070633	522.16	64980530	3688.47	2804950	187.66	69617736	3875.39
**1991**	9287653	521.27	66495711	3680.06	2854407	185.60	70871103	3844.00
**1992**	9502532	519.91	68047771	3672.18	2928520	184.90	72797292	3845.88
**1993**	9710835	518.25	69593550	3664.59	3053992	187.18	76059224	3914.12
**1994**	9915283	516.33	71145621	3656.90	3132414	186.38	78191134	3920.49
**1995**	10117470	514.34	72701192	3649.33	3172417	183.95	78968349	3870.93
**1996**	10313172	511.21	74372220	3643.36	3178878	179.87	78886227	3780.92
**1997**	10497595	506.55	76180475	3639.78	3189252	175.91	78957400	3696.76
**1998**	10678088	501.52	78055726	3637.66	3225897	173.42	79676915	3643.27
**1999**	10874742	497.14	80004356	3635.62	3289853	171.83	81385775	3625.04
**2000**	11099317	494.32	81957611	3632.50	3351438	170.00	82933058	3600.00
**2001**	11370253	493.22	83971965	3626.92	3417594	168.62	84316589	3570.84
**2002**	11675184	493.01	86044195	3619.95	3504176	168.12	86252080	3561.48
**2003**	11993762	493.15	88133655	3612.68	3604282	168.56	88308179	3560.07
**2004**	12319360	493.06	90319175	3605.82	3647202	165.84	89220753	3504.36
**2005**	12632594	492.11	92585298	3600.77	3722419	164.26	90926765	3477.92
**2006**	12924773	489.42	95104526	3598.11	3718310	159.20	90735264	3377.02
**2007**	13193573	485.16	97820571	3596.79	3789162	157.47	92076705	3335.95
**2008**	13449188	480.34	100598564	3595.81	3894382	156.95	94242823	3323.16
**2009**	13726373	476.11	103483165	3594.80	3954608	154.72	95133111	3266.97
**2010**	14044387	473.50	106350985	3593.47	4068597	154.39	97369097	3255.93
**2011**	14391760	471.71	109321607	3591.17	4147954	152.51	98962796	3219.48
**2012**	14733919	469.39	112427219	3587.29	4236672	150.91	100780344	3188.41
**2013**	15073498	466.89	115599384	3583.16	4339637	150.44	102277090	3155.68
**2014**	15426522	464.71	118919526	3579.71	4430653	149.41	103654540	3118.43
**2015**	15804295	463.31	122317745	3579.22	4483229	146.47	104781612	3067.00
**2016**	16202529	462.17	126018674	3582.62	4566910	144.40	106603007	3035.31
**2017**	16592135	460.50	129890361	3586.89	4622499	141.54	107514536	2980.00
**2018**	16985404	458.76	133874379	3591.86	4733082	140.28	109801277	2961.11
**2019**	17413449	457.68	138037016	3598.05	4840901	139.04	111816479	2936.29
**2020**	17746812	455.21	141868630	3603.24	4919961	137.66	113147786	2903.25
**2021**	17961630	450.39	145307703	3610.24	5002681	136.84	114982354	2890.65

Table S2 The number of prevalence, incidence, deaths, and DALYs and corresponding age-standardized rates in female IHD patients worldwide, from 1990 to 2021.

Year	Incidence		Prevalence		Deaths		DALYs	
	Number	Rate	Number	Rate	Number	Rate	Number	Rate
**1990**	6742985	329.74	47188959	2250.59	2562186	134.50	49545221	2407.52
**1991**	6895044	329.16	48199831	2246.25	2592752	132.29	50060847	2374.01
**1992**	7041823	328.24	49217046	2241.83	2647461	131.46	51055213	2364.15
**1993**	7187623	327.28	50241761	2238.02	2752601	133.12	52923565	2395.04
**1994**	7337515	326.42	51306566	2235.32	2810131	132.32	54043008	2388.94
**1995**	7487487	325.48	52382799	2232.46	2836028	130.18	54404967	2351.16
**1996**	7641395	324.45	53545832	2231.39	2839455	127.08	54431942	2298.78
**1997**	7792071	322.81	54789490	2231.24	2844269	123.99	54564990	2249.80
**1998**	7944538	321.06	56092731	2232.15	2868394	121.79	54947530	2211.96
**1999**	8106375	319.53	57463058	2233.82	2931452	121.16	56044725	2201.79
**2000**	8271331	317.99	58847135	2234.06	2951178	118.58	56436306	2162.56
**2001**	8447076	316.62	60288945	2233.25	2993335	116.93	57183587	2136.55
**2002**	8621907	315.02	61741500	2230.80	3065785	116.49	58443104	2128.96
**2003**	8796691	313.38	63192390	2227.74	3127875	115.66	59427884	2111.74
**2004**	8985768	311.95	64723075	2225.37	3125160	112.26	59372769	2055.34
**2005**	9180092	310.42	66305833	2223.25	3171579	110.54	60108619	2026.51
**2006**	9371586	308.32	68046582	2222.64	3160863	106.87	59833910	1963.19
**2007**	9548207	305.39	69907415	2222.62	3193366	104.73	60190892	1921.34
**2008**	9717244	302.22	71802145	2222.58	3260024	103.70	61120354	1898.07
**2009**	9910658	299.61	73802024	2223.30	3297560	101.65	61595266	1860.01
**2010**	10140931	298.01	75853805	2224.46	3360723	100.33	62575683	1837.45
**2011**	10401083	297.02	78017277	2225.72	3418097	98.82	63456789	1811.40
**2012**	10660147	295.77	80248187	2225.52	3481761	97.49	64718946	1795.61
**2013**	10919602	294.48	82509611	2225.16	3551641	96.40	65733621	1773.38
**2014**	11199358	293.60	84943437	2226.89	3610432	95.01	66636870	1747.90
**2015**	11506637	293.37	87520732	2231.99	3651289	93.19	67454961	1721.46
**2016**	11861580	294.02	90529554	2244.44	3709821	91.79	68571755	1702.23
**2017**	12246430	295.10	93859615	2261.55	3739772	89.69	68848735	1662.09
**2018**	12643871	296.29	97271042	2278.58	3815265	88.73	70244262	1649.56
**2019**	13054184	297.43	100659184	2291.77	3873814	87.35	71211910	1626.43
**2020**	13452279	298.66	103721839	2299.79	3919420	85.94	72017093	1603.44
**2021**	13911148	301.57	108968565	2357.61	3988956	85.32	73378204	1596.14

Table S3 The global number of prevalence, incidence, deaths, and DALYs of IHD and their rates by age distribution.

Year	Incidence		Prevalence		Deaths		DALYs	
	Number	Rate	Number	Rate	Number	Rate	Number	Rate
**15-19**	23629.36	3.79	81598.24	13.08	9161.36	1.47	668022.33	107.06
**20-24**	77990.24	13.06	229591.24	38.45	17878.77	2.99	1222528.53	204.72
**25-29**	142831.76	24.28	639618.16	108.72	28770.08	4.89	1830416.09	311.11
**30-34**	325661.12	53.87	1594163.74	263.72	58043.35	9.60	3393809.10	561.44
**35-39**	560612.23	99.95	3260621.41	581.35	91659.24	16.34	4903530.12	874.28
**40-44**	1038525.60	207.60	5867478.10	1172.91	159466.31	31.88	7733697.21	1545.96
**45-49**	1732119.24	365.81	10907131.73	2303.49	244829.80	51.71	10691370.11	2257.92
**50-54**	2586272.31	581.29	18242824.02	4100.22	379042.01	85.19	14731768.92	3311.08
**55-59**	3409516.47	861.58	26160210.14	6610.65	558748.23	141.20	19071783.47	4819.42
**60-64**	3842860.22	1200.71	31914240.93	9971.71	725717.37	226.75	21411394.96	6690.06
**65-69**	4222926.22	1530.92	41686590.26	15112.48	944626.53	342.45	23627678.53	8565.65
**70-74**	4061055.62	1972.93	39861593.80	19365.40	1100664.33	534.72	22710982.95	11033.36
**75-79**	3333991.71	2527.97	31024122.88	23523.72	1126256.12	853.97	18482164.87	14013.91
**80-84**	3055761.51	3488.99	23411541.34	26730.67	1347764.88	1538.84	17192462.58	19629.90
**85-89**	2062420.49	4510.80	12756877.67	27901.09	1172374.39	2564.15	11850738.30	25919.23
**90-94**	1022963.90	5718.28	5101418.86	28516.48	731620.88	4089.70	6411579.52	35840.16
**95 plus**	373640.16	6855.39	1536645.32	28193.73	295013.02	5412.78	2426629.75	44522.79

Table S4 The age-standardized rates and EAPC of prevalence, incidence, deaths, and DALYs for IHD in both sexes across 204 countries in 2021.

location	Incidence		Prevalence		Death		DALY	
	ASR, per 100,000, 2021	EAPC, %, 1990-2021	ASR, per 100,000, 2021	EAPC, %, 1990-2021	ASR, per 100,000, 2021	EAPC, %, 1990-2021	ASR, per 100,000, 2021	EAPC, %, 1990-2021
**Afghanistan**	964.61 (833.22,1125.98)	-0.78 (-0.85,-0.7)	6317.65 (5785.3,6886.79)	-0.3 (-0.34,-0.27)	280.03 (221.41,346.2)	-0.98 (-1.08,-0.89)	6178.27 (4747.55,7813.31)	-1.2 (-1.31,-1.09)
**Albania**	322.15 (263.65,390.87)	-0.04 (-0.12,0.04)	2943.69 (2607.84,3312.44)	-0.22 (-0.26,-0.18)	158.29 (134.2,185.54)	0.09 (-0.12,0.3)	2558.27 (2184.88,2998.29)	-0.34 (-0.54,-0.14)
**Algeria**	861.13 (753.02,1009.41)	-1.14 (-1.25,-1.02)	6265.94 (5755.97,6836.74)	-0.34 (-0.38,-0.3)	212.02 (172.72,251.71)	-1.21 (-1.26,-1.16)	3451.99 (2806.95,4159.87)	-1.68 (-1.77,-1.59)
**American Samoa**	403.26 (312.75,519.22)	0.2 (0.18,0.21)	3189.2 (2870.61,3544.63)	0.14 (0.13,0.14)	178.99 (155.05,207.56)	0.37 (0.32,0.43)	4053.34 (3456.83,4752.36)	0.34 (0.27,0.41)
**Andorra**	158.24 (125.36,201.39)	-0.73 (-0.81,-0.65)	1447.15 (1267.07,1640.49)	-0.58 (-0.65,-0.5)	41.02 (30.25,52.92)	-2.06 (-2.29,-1.83)	724.42 (539.1,929.5)	-2.28 (-2.51,-2.05)
**Angola**	346.95 (282.03,418.75)	-0.5 (-0.55,-0.45)	2316.3 (2069.3,2609.2)	-0.14 (-0.16,-0.12)	125.48 (99.59,156.4)	-0.24 (-0.32,-0.15)	2529.04 (1954.37,3169.14)	-0.44 (-0.52,-0.36)
**Antigua and Barbuda**	382.38 (302.88,481.55)	0.13 (0.1,0.16)	3347.62 (2995.31,3737.23)	0.24 (0.23,0.25)	71.43 (66.59,77.89)	-2.17 (-2.39,-1.96)	1306.35 (1226.11,1441.33)	-2.49 (-2.69,-2.28)
**Argentina**	243.87 (207.22,291.57)	-1.61 (-1.88,-1.34)	1666.56 (1499.65,1871.35)	-0.73 (-0.82,-0.64)	60.79 (55.95,64.11)	-2.68 (-2.86,-2.5)	1179.87 (1113.64,1234.14)	-2.74 (-2.9,-2.59)
**Armenia**	591 (490.42,710.55)	-0.49 (-0.56,-0.42)	4081.36 (3674.11,4505.4)	0.25 (0.22,0.28)	209.78 (186.39,233.39)	-1.98 (-2.2,-1.75)	3788.46 (3403.75,4233.65)	-1.96 (-2.15,-1.76)
**Australia**	213.72 (176.25,262.25)	-1.43 (-1.8,-1.06)	1877.69 (1685.68,2103.19)	-0.65 (-0.84,-0.45)	44.08 (37.95,47.53)	-4.68 (-4.79,-4.57)	768.62 (696.1,814.23)	-4.78 (-4.92,-4.64)
**Austria**	192.62 (170.88,218.82)	-0.74 (-0.81,-0.67)	1647.51 (1470.77,1832.73)	0.22 (0.17,0.26)	68.11 (59.02,73.18)	-3.16 (-3.35,-2.98)	1121.64 (1017.81,1189.7)	-3.54 (-3.76,-3.33)
**Azerbaijan**	806.26 (725.6,906.9)	1.02 (0.88,1.17)	4415.23 (4047.75,4805.9)	0.36 (0.32,0.39)	306.13 (270.78,343.48)	-0.37 (-0.57,-0.18)	5496.28 (4818.68,6182.32)	-0.95 (-1.17,-0.74)
**Bahamas**	401.65 (318.36,502.75)	0.12 (0.1,0.15)	3508.61 (3146.7,3904.39)	0.25 (0.23,0.27)	76.31 (63.63,92.15)	-1.89 (-2.08,-1.7)	1624.95 (1339.72,1979.27)	-1.97 (-2.15,-1.78)
**Bahrain**	1023.47 (792.34,1294.14)	-0.47 (-0.55,-0.4)	7066.05 (6420.79,7782.27)	-0.19 (-0.23,-0.15)	161.62 (140.64,184.38)	-3.56 (-3.94,-3.18)	2796.56 (2418.62,3179.47)	-3.98 (-4.31,-3.64)
**Bangladesh**	469.75	-0.15	3969.58	0.16	107.5	-0.19	2366.92	-0.55

location	Incidence		Prevalence		Death		DALY	
	ASR, per 100,000, 2021	EAPC, %, 1990-2021	ASR, per 100,000, 2021	EAPC, %, 1990-2021	ASR, per 100,000, 2021	EAPC, %, 1990-2021	ASR, per 100,000, 2021	EAPC, %, 1990-2021
	(405.86,551.3)	(-0.21,-0.08)	(3596.91,4375.69)	(0.12,0.2)	(86.2,131.71)	(-0.33,-0.06)	(1856.38,2929.73)	(-0.63,-0.46)
**Barbados**	390.55	0.21	3422.85	0.35	62.55	-2.26	1150.8	-2.32
	(308.04,491.17)	(0.17,0.25)	(3064.35,3832)	(0.3,0.4)	(51.4,74.63)	(-2.57,-1.95)	(936.24,1387.58)	(-2.61,-2.03)
**Belarus**	606.69	0.03	3891.64	0.11	341.51	-0.3	6244.29	-0.42
	(552.13,679.03)	(-0.16,0.22)	(3553.03,4226.16)	(0.02,0.21)	(292.04,394.62)	(-0.61,0.02)	(5262.84,7270.21)	(-0.83,0)
**Belgium**	170.49	-2.5	1432.88	-1.27	38.04	-4.09	698.85	-4.17
	(138.34,207.13)	(-2.96,-2.03)	(1254.81,1632.08)	(-1.46,-1.09)	(33.11,40.92)	(-4.24,-3.94)	(638.41,740.15)	(-4.3,-4.05)
**Belize**	378.41	0.19	3219.61	0.37	66.97	-2.42	1363.17	-2.51
	(294.1,474.76)	(0.16,0.22)	(2885.16,3604.55)	(0.35,0.39)	(59.34,74.28)	(-2.77,-2.06)	(1223.34,1513.59)	(-2.84,-2.18)
**Benin**	361.71	-0.12	2467.27	0.25	77.65	0.24	1514.95	0.12
	(286.18,449.82)	(-0.16, 0.08)	(2189.84,2771.31)	(0.22,0.27)	(65.25,93.07)	(0.08,0.41)	(1244.52,1848.81)	(-0.06,0.3)
**Bermuda**	373.73	0.05	3439.03	0.25	68.35	-4.11	1283.78	-4.12
	(294.5,470.34)	(0.01,0.09)	(3079.38,3832.95)	(0.23,0.26)	(59.38,81.13)	(-4.44,-3.78)	(1113.11,1520.67)	(-4.49,-3.76)
**Bhutan**	587.35	-0.03	4367.48	0.19	110.98	0.2	2299.54	-0.25
	(453.84,739.37)	(-0.05,-0.01)	(3913.37,4869.9)	(0.18,0.2)	(86.52,133.92)	(0.17,0.24)	(1757.99,2871.27)	(-0.28,-0.23)
**Bolivia (Plurinational State of)**	251.69	-0.08	2120.91	0.22	84.63	-1.6	1602.36	-1.86
	(203.87,304.63)	(-0.13,-0.03)	(1891.18,2372.51)	(0.19,0.26)	(62.4,121.7)	(-1.83,-1.37)	(1154.23,2291.24)	(-2.1,-1.61)
**Bosnia and Herzegovina**	324.88	-0.59	3204.01	0.11	138.3	-1.6	2450.2	-1.95
	(265.57,394.41)	(-0.68,-0.51)	(2832.71,3582.71)	(0.07,0.15)	(114.1,162.53)	(-1.9,-1.3)	(2019.97,2859.4)	(-2.2,-1.7)
**Botswana**	379.05	0.13	2743.35	0.36	78.6	-0.86	1574.31	-1.06
	(294.21,473.61)	(0.09,0.17)	(2440.89,3073.51)	(0.33,0.4)	(62.09,98.66)	(-1.12,-0.6)	(1216.62,1984.6)	(-1.36,-0.76)
**Brazil**	167.57	-0.37	1976.74	0.07	64.01	-2.32	1469.72	-2.16
	(136.27,200.4)	(-0.45,-0.3)	(1669.26,2325.71)	(0.04,0.1)	(58.63,67.35)	(-2.41,-2.22)	(1380.14,1530.27)	(-2.23,-2.09)
**Brunei Darussalam**	115.47	-0.36	937.69	-0.69	84.5	-1.42	1726.32	-1.69
	(90.12,146.75)	(-0.41,-0.32)	(832.55,1056.19)	(-0.75,-0.64)	(74.32,95.88)	(-1.66,-1.17)	(1525.62,1929.94)	(-1.92,-1.47)
**Bulgaria**	413.13	-1.49	3321.73	-0.36	204.4	-2.85	3852.25	-2.51
	(364.08,471.42)	(-1.73,-1.24)	(2961.85,3660.73)	(-0.43,-0.3)	(180.5,230.3)	(-3.18,-2.51)	(3333.94,4409.66)	(-2.82,-2.19)
**Burkina Faso**	336.69	0	2238.55	0.14	97.57	0.75	1884.27	0.59
	(273.26,411.12)	(-0.03,0.03)	(1979.61,2508.54)	(0.09,0.2)	(75.51,124.07)	(0.61,0.89)	(1448.29,2432.51)	(0.48,0.7)
**Burundi**	317.54	-0.24	2091.39	-0.03	85.8	-1.17	1841.44	-1.34
	(247.49,398.05)	(-0.28,-0.2)	(1846.86,2372.87)	(-0.04,-0.01)	(68,108.32)	(-1.36,-0.98)	(1452.49,2345.53)	(-1.55,-1.14)

location	Incidence		Prevalence		Death		DALY	
	ASR, per 100,000, 2021	EAPC, %, 1990-2021	ASR, per 100,000, 2021	EAPC, %, 1990-2021	ASR, per 100,000, 2021	EAPC, %, 1990-2021	ASR, per 100,000, 2021	EAPC, %, 1990-2021
**Cabo Verde**	391.91 (307.85,495.61)	0.18 (0.14,0.22)	2813.85 (2504.58,3182.1)	0.46 (0.44,0.48)	121.51 (100.46,142.26)	1.14 (0.76,1.52)	2236.65 (1830.13,2642.4)	0.83 (0.5,1.17)
**Cambodia**	237.77 (197.74,281.58)	0.37 (0.27,0.47)	1828.62 (1645.61,2037.78)	0.3 (0.25,0.35)	111.55 (89.23,134.56)	-0.17 (-0.26,-0.09)	2251.18 (1767.77,2790.06)	-0.58 (-0.66,-0.5)
**Cameroon**	340.42 (281.49,413.15)	0.12 (0.01,0.23)	2276.99 (2033.42,2565.31)	0.39 (0.3,0.48)	103.29 (79.89,137.12)	0.95 (0.41,1.49)	2044.68 (1551.58,2747.31)	0.93 (0.35,1.51)
**Canada**	204.24 (170.67,245.09)	-2.65 (-2.89,-2.41)	1544.09 (1400.94,1734.92)	-1.48 (-1.62,-1.34)	51.12 (45.43,54.44)	-3.88 (-4.03,-3.73)	922.52 (849.26,968.23)	-3.88 (-4.03,-3.74)
**Central African Republic**	387.14 (305,487.17)	-0.05 (-0.08,-0.02)	2163.54 (1902.93,2463.37)	-0.04 (-0.06,-0.02)	150.26 (109.43,210.57)	-0.42 (-0.5,-0.35)	3256.9 (2364.11,4604.4)	-0.48 (-0.57,-0.4)
**Chad**	375.09 (298.44,461.03)	-0.06 (-0.1,-0.02)	2623.66 (2319.45,2950.56)	0.31 (0.29,0.34)	104.29 (80.34,131.28)	0.47 (0.26,0.68)	2128.18 (1619.46,2717.98)	0.47 (0.24,0.71)
**Chile**	108.52 (97.55,128.5)	-1.56 (-1.88,-1.24)	1273.73 (1143.98,1434.25)	-0.36 (-0.42,-0.29)	39.05 (35.77,41.32)	-3.41 (-3.53,-3.28)	808.36 (760.79,847.46)	-2.9 (-3.01,-2.8)
**China**	365.67 (293.32,440.07)	0.66 (0.51,0.82)	3042.35 (2601.68,3629.87)	0.64 (0.56,0.72)	110.91 (92.42,128.56)	0.97 (0.64,1.29)	1856.51 (1548.73,2159.82)	0.51 (0.25,0.77)
**Colombia**	261.03 (218.32,311.76)	-0.93 (-1.02,-0.84)	2298.99 (2053.29,2544.74)	-0.45 (-0.5,-0.4)	79.05 (66.64,93.24)	-2.13 (-2.32,-1.93)	1502.23 (1264.42,1754.08)	-2.38 (-2.58,-2.17)
**Comoros**	335.59 (264.21,426.51)	0.01 (0,0.03)	2443.24 (2160.72,2775.85)	0.07 (0.04,0.1)	73.16 (55.29,95.05)	-0.44 (-0.56,-0.33)	1526.07 (1137.18,1995.75)	-0.64 (-0.79,-0.48)
**Congo**	391.55 (307.1,482.59)	-0.08 (-0.1,-0.05)	2522.81 (2243.21,2861.25)	0.15 (0.12,0.18)	158.95 (127.71,189.82)	-0.8 (-0.93,-0.67)	3210.45 (2515.58,3995.7)	-1.03 (-1.18,-0.87)
**Cook Islands**	381.65 (297.16,486.76)	0.3 (0.28,0.32)	3134.55 (2841.95,3485.26)	0.34 (0.33,0.35)	107.15 (88.99,128.05)	-1.25 (-1.37,-1.13)	2344.82 (1931.57,2837.91)	-1.23 (-1.38,-1.07)
**Costa Rica**	325.92 (259.79,401.55)	-0.43 (-0.48,-0.38)	2986.49 (2672.05,3332.15)	-0.07 (-0.09,-0.05)	55.1 (48.17,61.1)	-2.65 (-2.82,-2.48)	1157.79 (1033.42,1276.35)	-2.42 (-2.61,-2.24)
**Coted'Ivoire**	390.73 (316.46,467.78)	-0.21 (-0.27,-0.15)	2737.65 (2446.21,3057.16)	0.16 (0.14,0.17)	119.9 (98.75,152.67)	-0.06 (-0.29,0.17)	2390.53 (1895.8,3151.33)	-0.16 (-0.41,0.08)
**Croatia**	245.54 (213.01,283.69)	-1.55 (-2.02,-1.08)	2776.1 (2434.27,3154.31)	-0.44 (-0.59,-0.3)	130.52 (114.93,143.14)	-2.6 (-2.72,-2.47)	2099.58 (1856.5,2326.01)	-2.76 (-2.85,-2.68)
**Cuba**	330.27	-1.11	2976.59	-0.43	104.76	-2.56	2017.01	-2.43

location	Incidence		Prevalence		Death		DALY	
	ASR, per 100,000, 2021	EAPC, %, 1990-2021	ASR, per 100,000, 2021	EAPC, %, 1990-2021	ASR, per 100,000, 2021	EAPC, %, 1990-2021	ASR, per 100,000, 2021	EAPC, %, 1990-2021
	(274.53,396.4)	(-1.34,-0.88)	(2669.05,3331.59)	(-0.58,-0.28)	(91.34,117.29)	(-2.86,-2.27)	(1763.5,2255.67)	(-2.71,-2.14)
**Cyprus**	119.58	-1.31	1198.39	-0.77	86.95	-4.03	1394.96	-3.81
	(95.44,148.28)	(-2.03,-0.59)	(1033.8,1385.58)	(-1.01,-0.53)	(75.85,98.11)	(-4.23,-3.83)	(1225.32,1564.95)	(-3.96,-3.66)
**Czechia**	369.33	-1.74	3350.08	-0.91	126.68	-2.56	2101.42	-3.16
	(314.8,435.61)	(-2.04,-1.43)	(2991.38,3711.04)	(-1.07,-0.75)	(110.35,139.62)	(-2.66,-2.45)	(1857.11,2327.15)	(-3.27,-3.06)
**Democratic People's Republic of Korea**	346.24	0.15	2631.7	0.13	125.35	0.38	2594.23	0.45
	(293.67,411.15)	(0.07,0.22)	(2381.97,2915.77)	(0.11,0.14)	(100.79,153.33)	(0.19,0.56)	(2058.22,3211.81)	(0.27,0.63)
**Democratic Republic of the Congo**	336.17	-0.43	2055.21	-0.36	112.32	-0.65	2290.38	-0.67
	(285.3,397.27)	(-0.47,-0.39)	(1840.49,2308.83)	(-0.4,-0.32)	(83.28,149.12)	(-0.74,-0.55)	(1696.29,3056.52)	(-0.77,-0.58)
**Denmark**	132.46	-0.73	1085.82	-0.4	39.95	-5.83	710.23	-5.81
	(107.93,164)	(-1.03,-0.43)	(948.09,1240.27)	(-0.49,-0.31)	(34.9,42.81)	(-5.99,-5.66)	(653.57,756)	(-6,-5.63)
**Djibouti**	359.64	0.23	2668.37	0.36	91.49	0.8	1927.16	0.71
	(278.64,456.56)	(0.22,0.24)	(2349.24,3018.09)	(0.34,0.39)	(67.7,118.94)	(0.67,0.94)	(1417.07,2555.52)	(0.56,0.87)
**Dominica**	384.71	0.14	3198.26	0.29	92.21	-1.81	1738.9	-1.65
	(299.19,481.6)	(0.1,0.18)	(2863.71,3587.49)	(0.25,0.32)	(81.49,107.84)	(-2.07,-1.55)	(1513.77,2064.5)	(-1.93,-1.37)
**Dominican Republic**	383.62	0.55	3246.16	0.49	144.66	0.54	3104.02	0.71
	(323.09,448.03)	(0.45,0.65)	(2931.53,3606.37)	(0.45,0.53)	(116.75,177.94)	(0.31,0.77)	(2494.68,3824.78)	(0.54,0.88)
**Ecuador**	250.34	-0.02	2312.92	0.29	77.47	-0.89	1433.67	-1.02
	(204.21,301.14)	(-0.06,0.02)	(2056.89,2582.21)	(0.28,0.31)	(64.11,93.12)	(-1.44,-0.34)	(1158.9,1770.44)	(-1.53,-0.52)
**Egypt**	1063.03	0.19	7100.29	0.45	347.73	0.2	6924.84	0.07
	(927.44,1200.31)	(0.09,0.28)	(6580.94,7664.24)	(0.42,0.48)	(297.31,402.14)	(-0.01,0.41)	(5844.94,8119.21)	(-0.12,0.25)
**El Salvador**	293.73	0.03	2380.22	0.28	94.74	-0.79	1915.93	-0.93
	(237,360.88)	(0.01,0.05)	(2132.17,2657.84)	(0.26,0.3)	(78.35,113.9)	(-0.99,-0.58)	(1577.46,2311.23)	(-1.18,-0.68)
**Equatorial Guinea**	381.85	0.01	2663.51	0.67	141.8	-0.57	2751.44	-0.99
	(298.35,486.93)	(-0.04,0.06)	(2380.36,3001.56)	(0.6,0.74)	(102.97,188.38)	(-0.77,-0.37)	(1907.88,3773.97)	(-1.24,-0.74)
**Eritrea**	310.99	-0.14	2044.55	0.09	90.95	0.28	1974.98	0.06
	(243.09,391.38)	(-0.17,-0.1)	(1794.84,2337.1)	(0.07,0.11)	(69.21,117.14)	(0.2,0.35)	(1470.4,2570.23)	(-0.01,0.13)
**Estonia**	559.35	-0.64	3966.29	0.28	92.06	-4.84	1547.87	-5.13
	(451.61,686.34)	(-0.83,-0.46)	(3592.13,4385.34)	(0.17,0.39)	(80.72,102.43)	(-5.25,-4.43)	(1370.4,1716.96)	(-5.54,-4.73)
**Eswatini**	365.62	0.22	2532.5	0.3	106.44	1.06	2334.11	1.21
	(284.58,461.07)	(0.2,0.24)	(2240.98,2848.72)	(0.25,0.36)	(75.27,145.87)	(0.55,1.58)	(1612.79,3342.38)	(0.6,1.82)

location	Incidence		Prevalence		Death		DALY	
	ASR, per 100,000, 2021	EAPC, %, 1990-2021	ASR, per 100,000, 2021	EAPC, %, 1990-2021	ASR, per 100,000, 2021	EAPC, %, 1990-2021	ASR, per 100,000, 2021	EAPC, %, 1990-2021
**Ethiopia**	286.9 (225.65,356.63)	-0.52 (-0.61,-0.43)	1991.85 (1665.71,2376.33)	-0.01 (-0.07,0.05)	55.81 (45.21,66.72)	-1.51 (-1.62,-1.4)	1177.38 (959.27,1410.32)	-1.91 (-2.04,-1.78)
**Fiji**	441.76 (346.45,549.38)	0.09 (0.03,0.15)	3404.98 (3076.64,3782.55)	0.13 (0.11,0.15)	266.78 (216.97,322.31)	-0.5 (-0.64,-0.35)	5965.61 (4721.09,7391.81)	-0.64 (-0.75,-0.53)
**Finland**	224.32 (187.44,269.62)	-2.39 (-3,-1.78)	1618.74 (1418.61,1836.71)	-1.49 (-1.81,-1.16)	77.38 (65.63,83.77)	-3.28 (-3.35,-3.22)	1299.12 (1163.23,1383.11)	-3.77 (-3.81,-3.73)
**France**	176.27 (147.06,213.19)	-0.98 (-1.19,-0.78)	1595.12 (1425.27,1801.5)	-0.78 (-0.88,-0.68)	29.87 (25.89,32.3)	-3.48 (-3.6,-3.36)	555.98 (503.45,597.26)	-3.36 (-3.46,-3.26)
**Gabon**	366.64 (286,458.79)	0.15 (0.14,0.15)	2482.72 (2217.3,2808.99)	0.23 (0.22,0.24)	126.77 (99.33,155.15)	-0.33 (-0.45,-0.21)	2479.16 (1901.2,3118.09)	-0.46 (-0.58,-0.34)
**Gambia**	416.26 (325.28,522.86)	0.02 (-0.01,0.04)	2794.08 (2490.2,3145.69)	0.17 (0.15,0.19)	140.02 (108.58,171.76)	0.61 (0.47,0.75)	2771.5 (2116.36,3471.6)	0.5 (0.33,0.67)
**Georgia**	553.85 (458.86,662.12)	-1.49 (-1.6,-1.37)	4117.67 (3704.65,4551.63)	-0.35 (-0.39,-0.31)	124.16 (111.04,136.51)	-4.01 (-4.41,-3.61)	2565.24 (2301.9,2822.2)	-4.04 (-4.5,-3.58)
**Germany**	223.88 (195.43,261.15)	-2.34 (-2.45,-2.24)	1683.17 (1496,1900.56)	-1.28 (-1.33,-1.22)	63.67 (55.51,68.32)	-3.72 (-3.84,-3.61)	1096.63 (993.52,1160.04)	-3.86 (-3.97,-3.74)
**Ghana**	387.94 (322.49,459.15)	-0.48 (-0.56,-0.41)	2706.34 (2416.5,3020.02)	0.12 (0.08,0.16)	97.56 (78.35,118.19)	-1.76 (-2.09,-1.43)	1954.33 (1558.46,2392.96)	-1.84 (-2.17,-1.51)
**Greece**	124.49 (108.66,140.87)	-1.99 (-2.12,-1.86)	1157.57 (1019.15,1318.79)	-1.02 (-1.1,-0.94)	72.9 (65.2,76.93)	-2.33 (-2.64,-2.02)	1443.35 (1353.57,1503.27)	-2.09 (-2.32,-1.86)
**Greenland**	223.4 (175.22,284.24)	-0.52 (-0.59,-0.45)	1650.3 (1474.68,1871.57)	-0.15 (-0.21,-0.1)	81.2 (70.57,97.64)	-3.17 (-3.34,-2.99)	1632.37 (1432.87,1918.7)	-3.18 (-3.34,-3.03)
**Grenada**	395.82 (313.85,493.35)	0.17 (0.12,0.22)	3305.1 (2940.5,3698.39)	0.34 (0.3,0.38)	95.36 (83.86,105.78)	-1.84 (-2.14,-1.55)	1924.27 (1678.96,2155)	-1.98 (-2.26,-1.7)
**Guam**	381.86 (301.27,479.77)	0.63 (0.6,0.67)	3116.71 (2804.27,3464.74)	0.58 (0.54,0.62)	126.6 (113.51,139.39)	-0.74 (-1.07,-0.4)	3413.08 (3110.68,3756.02)	0.12 (-0.13,0.36)
**Guatemala**	288.62 (236.43,349.71)	0.05 (-0.02,0.12)	2145.5 (1924.08,2397.53)	0.34 (0.32,0.37)	97.26 (85.34,109.04)	-1.36 (-1.77,-0.94)	1732.06 (1517.52,1959.37)	-1.67 (-2.17,-1.18)
**Guinea**	369.17 (299.07,447.29)	0.23 (0.2,0.26)	2442.44 (2155.68,2746.16)	0.41 (0.4,0.43)	110.41 (85.87,140.24)	0.84 (0.71,0.98)	2209.38 (1690.78,2864.84)	0.82 (0.68,0.96)
**Guinea-Bissau**	405.01	0.08	2525.64	0.26	156.92	0.2	3270.22	0.04

location	Incidence		Prevalence		Death		DALY	
	ASR, per 100,000, 2021	EAPC, %, 1990-2021	ASR, per 100,000, 2021	EAPC, %, 1990-2021	ASR, per 100,000, 2021	EAPC, %, 1990-2021	ASR, per 100,000, 2021	EAPC, %, 1990-2021
	(321.49,508.41)	(0.07,0.08)	(2246.27,2852.32)	(0.25,0.27)	(123.29,192.38)	(0.11,0.29)	(2536.54,4074.07)	(-0.05,0.14)
**Guyana**	397.67 (315.74,495.41)	-0.11 (-0.13,-0.08)	3163.78 (2832.6,3534.25)	0.11 (0.09,0.12)	143.23 (115.5,177.3)	-1.3 (-1.56,-1.03)	3075.23 (2425.76,3871.77)	-1.35 (-1.57,-1.14)
**Haiti**	401.8 (337.38,473.47)	-0.26 (-0.32,-0.2)	3053.58 (2724.06,3421.2)	0.04 (0.01,0.06)	210.11 (159.57,271.2)	-0.57 (-0.65,-0.48)	4370.88 (3271.69,5735.77)	-0.62 (-0.72,-0.53)
**Honduras**	365.94 (304.09,434.34)	0.5 (0.43,0.57)	2705.22 (2431.71,2980.8)	0.25 (0.24,0.27)	167.93 (139.63,201.92)	1.49 (1.25,1.74)	3075.05 (2550.8,3764.35)	1.23 (1.05,1.41)
**Hungary**	382.61 (333.99,448.41)	-1.3 (-1.36,-1.23)	3477.46 (3136.1,3845.7)	-1.04 (-1.11,-0.98)	155.36 (137.21,170.61)	-1.71 (-1.85,-1.58)	2780.98 (2470.6,3060.36)	-2.2 (-2.32,-2.08)
**Iceland**	188.79 (148.97,235.24)	-1.59 (-2.02,-1.16)	1563.07 (1378.97,1767.67)	-1.15 (-1.39,-0.91)	57.95 (49,64.16)	-3.42 (-3.52,-3.32)	990.46 (883.5,1083.79)	-3.73 (-3.79,-3.67)
**India**	585.23 (470.56,713.17)	-0.14 (-0.23,-0.06)	4458.18 (3744.48,5412.76)	0.27 (0.25,0.28)	151.17 (137.46,165.08)	0.48 (0.3,0.65)	3400.03 (3098.47,3720.83)	0.18 (0.06,0.3)
**Indonesia**	270.65 (215.27,331.46)	0.61 (0.51,0.71)	2349.89 (1998.83,2782.38)	0.5 (0.46,0.53)	143.25 (119.34,163.3)	1.19 (1.09,1.29)	3043.08 (2527.28,3544.71)	0.87 (0.78,0.96)
**Iran (Islamic Republic of)**	887.06 (698.93,1089.01)	-0.85 (-1.07,-0.63)	6327.23 (5393.23,7501.96)	-0.18 (-0.27,-0.1)	146.11 (130.71,156.95)	-2.08 (-2.22,-1.93)	2731.3 (2517.27,2920.87)	-2.26 (-2.4,-2.12)
**Iraq**	1040.55 (915.13,1202.99)	-0.07 (-0.12,-0.02)	7185.54 (6654.59,7885.02)	0.12 (0.1,0.13)	254.83 (204.17,296.68)	-0.43 (-0.62,-0.25)	4905.27 (3858.32,5827.68)	-0.84 (-0.98,-0.7)
**Ireland**	170.13 (136.02,208.82)	-2.64 (-2.88,-2.4)	1436.8 (1255.1,1625.39)	-1.42 (-1.57,-1.27)	52.77 (44.84,57.37)	-4.77 (-4.94,-4.59)	924.25 (829.87,991.72)	-5.04 (-5.21,-4.87)
**Israel**	153.19 (122.65,188.99)	-2.2 (-2.39,-2.01)	1435.4 (1255.57,1633.61)	-1.06 (-1.13,-0.98)	34.77 (29.93,37.49)	-5.69 (-5.89,-5.48)	599.57 (537.77,637.8)	-5.8 (-6.03,-5.57)
**Italy**	166.39 (138.51,194.88)	-1.71 (-1.8,-1.62)	1544.57 (1296.3,1834.07)	-0.88 (-0.96,-0.81)	44.27 (37.56,47.75)	-3.04 (-3.13,-2.95)	747.14 (673.73,791.65)	-3.32 (-3.4,-3.25)
**Jamaica**	368.87 (289.31,457.19)	0.09 (0.04,0.14)	3285.34 (2942.44,3675.94)	0.33 (0.29,0.38)	52.45 (42.06,66.21)	-0.64 (-1.14,-0.14)	1054.54 (837.96,1345.47)	-0.51 (-0.97,-0.04)
**Japan**	90.63 (71.46,113.97)	-0.3 (-0.46,-0.13)	802.34 (686.82,938.16)	-0.77 (-0.87,-0.67)	25.36 (21.7,27.29)	-2.95 (-3.18,-2.73)	502.25 (460.02,526.29)	-2.51 (-2.65,-2.37)
**Jordan**	913.43 (737.79,1134.18)	-0.52 (-0.63,-0.4)	6816.99 (6204.99,7546.39)	-0.02 (-0.04,0.01)	98.28 (80.73,117.66)	-2.83 (-3.14,-2.52)	1947.22 (1603.41,2349.61)	-3.09 (-3.4,-2.78)

location	Incidence		Prevalence		Death		DALY	
	ASR, per 100,000, 2021	EAPC, %, 1990-2021	ASR, per 100,000, 2021	EAPC, %, 1990-2021	ASR, per 100,000, 2021	EAPC, %, 1990-2021	ASR, per 100,000, 2021	EAPC, %, 1990-2021
**Kazakhstan**	584.39 (515.35,677.98)	-0.67 (-0.96,-0.38)	4230.88 (3828.87,4676.98)	-0.21 (-0.29,-0.13)	235.98 (212.04,258.87)	-1.88 (-2.44,-1.32)	3970.5 (3569.75,4401.12)	-2.5 (-3.14,-1.84)
**Kenya**	349.26 (271.66,443.54)	-0.04 (-0.07,-0.01)	2590.54 (2181.28,3081.36)	0.05 (0,0.1)	61.5 (46.7,79.16)	1.71 (1.45,1.97)	1244.93 (964.97,1595.88)	1.62 (1.3,1.94)
**Kiribati**	424.76 (331.65,544.55)	0.02 (-0.01,0.04)	3000.51 (2696.55,3362.27)	-0.04 (-0.05,-0.02)	202.94 (169.31,243.41)	0.24 (0.21,0.28)	4969.44 (4015.48,6139.26)	0.17 (0.12,0.21)
**Kuwait**	1061.15 (836.06,1330.52)	-0.3 (-0.41,-0.2)	7806.45 (7141.59,8556.31)	-0.03 (-0.09,0.02)	109.1 (90.44,131.44)	-1.89 (-2.3,-1.47)	2299.44 (1917.04,2762.48)	-1.95 (-2.37,-1.53)
**Kyrgyzstan**	594.1 (510.66,697.78)	0.17 (0.07,0.27)	3492.31 (3183.66,3858.39)	-0.21 (-0.24,-0.18)	274.41 (234.16,313.72)	0.36 (-0.01,0.72)	4780.48 (4093.58,5490.56)	-0.23 (-0.63,0.17)
**Lao People's Democratic Republic**	250.17 (205.22,302.23)	-0.03 (-0.12,0.06)	1968.29 (1773.75,2200.46)	0.05 (-0.02,0.12)	176.46 (141.65,213.26)	-1.07 (-1.17,-0.96)	3667.08 (2896.8,4573.18)	-1.44 (-1.54,-1.34)
**Latvia**	385.1 (320.59,460.54)	-1.26 (-1.58,-0.94)	3092.91 (2750.95,3480.56)	-0.56 (-0.7,-0.41)	160.3 (141.76,177.14)	-2.75 (-3.05,-2.45)	2916.55 (2581.61,3230.55)	-3.07 (-3.43,-2.71)
**Lebanon**	931.42 (756.1,1127.78)	-0.23 (-0.27,-0.18)	6814.65 (6242.72,7463.04)	0.26 (0.24,0.28)	92.09 (77.66,107.98)	-3.09 (-3.38,-2.79)	1735.3 (1488.38,2027.36)	-3.37 (-3.72,-3.01)
**Lesotho**	337.23 (262.89,422.87)	0.34 (0.32,0.37)	2086.28 (1849.58,2341.28)	0.49 (0.47,0.51)	90.9 (63.42,134.25)	2.84 (2.31,3.38)	1949.42 (1307.36,2989.62)	3.06 (2.5,3.63)
**Liberia**	397.86 (309.19,493.34)	-0.01 (-0.03,0.02)	2643.86 (2332.08,2977.66)	0.28 (0.25,0.31)	114.25 (90.14,145.7)	0.18 (0.05,0.31)	2264 (1760.67,2953.76)	0.16 (0.03,0.29)
**Libya**	922.52 (746.18,1115.01)	-0.02 (-0.1,0.06)	6634.68 (6059.17,7302.98)	0.1 (0.06,0.13)	178.47 (140.3,228.13)	0.83 (0.59,1.07)	3765.46 (2952.85,4839.08)	0.68 (0.46,0.89)
**Lithuania**	413.59 (360.41,476.27)	-0.68 (-1,-0.36)	3040.21 (2715.44,3402.97)	-0.28 (-0.44,-0.12)	197.08 (176.61,215.91)	-1.97 (-2.19,-1.74)	3332.3 (3000.98,3676.82)	-2.2 (-2.48,-1.92)
**Luxembourg**	117.08 (91.6,147.19)	-0.65 (-1.35,0.06)	1239.81 (1079.44,1411.72)	-0.31 (-0.47,-0.16)	45.7 (40.6,50.17)	-4.01 (-4.16,-3.86)	789.76 (717.69,867.8)	-4.29 (-4.46,-4.13)
**Madagascar**	346.21 (282.01,415.54)	-0.04 (-0.07,-0.01)	2382.91 (2115.55,2683.59)	0.16 (0.16,0.17)	100.02 (74.95,125.89)	0.27 (0.12,0.41)	2205.88 (1634.85,2810.29)	0.17 (0.03,0.31)
**Malawi**	335.7 (268.96,412.55)	-0.2 (-0.27,-0.12)	2429.14 (2141.61,2767.89)	0.1 (0.03,0.17)	84.27 (70.14,99.95)	0.39 (0.13,0.66)	1869.9 (1556.4,2221.41)	0.37 (0.07,0.67)
**Malaysia**	313.1	-0.09	2857.76	0.52	149.66	-0.64	3180.36	-0.68

location	Incidence		Prevalence		Death		DALY	
	ASR, per 100,000, 2021	EAPC, %, 1990-2021	ASR, per 100,000, 2021	EAPC, %, 1990-2021	ASR, per 100,000, 2021	EAPC, %, 1990-2021	ASR, per 100,000, 2021	EAPC, %, 1990-2021
	(271.03,364.9)	(-0.2,0.02)	(2581.66,3143.7)	(0.46,0.57)	(138.24,159.74)	(-0.8,-0.49)	(2984.07,3352.24)	(-0.79,-0.56)
**Maldives**	221.43	-0.26	2050.89	-0.26	79.43	-2.8	1481.02	-3.5
	(173.67,279)	(-0.34,-0.19)	(1821.79,2311.71)	(-0.31,-0.21)	(66.25,93.77)	(-2.91,-2.69)	(1238.38,1759.98)	(-3.66,-3.35)
**Mali**	351.99	-0.17	2298.64	0.23	71.65	-0.04	1439.78	-0.12
	(284.45,433.23)	(-0.22,-0.13)	(2040.33,2607.61)	(0.21,0.26)	(56.24,90.99)	(-0.14,0.06)	(1125.23,1851.95)	(-0.22,-0.01)
**Malta**	121.22	-1.06	1272.1	-0.51	71.19	-3.43	1270.51	-3.53
	(94.34,152.42)	(-1.69,-0.42)	(1103.48,1448.13)	(-0.69,-0.32)	(61.3,78.48)	(-3.62,-3.23)	(1140.12,1387.85)	(-3.69,-3.37)
**Marshall Islands**	381.21	0.11	2756.98	0.12	275.9	0.23	6518.18	0.26
	(298.36,483.58)	(0.09,0.14)	(2461.12,3083.53)	(0.1,0.15)	(219.83,337.91)	(0.12,0.35)	(5093.82,8205.42)	(0.13,0.38)
**Mauritania**	398.48	-0.17	2847.47	0.22	112.23	-0.95	2126.35	-1.16
	(311.12,498.68)	(-0.21,-0.12)	(2543.25,3209.18)	(0.2,0.25)	(85.32,144.29)	(-1.14,-0.76)	(1570.63,2744.37)	(-1.34,-0.97)
**Mauritius**	224.16	-1.12	2124.59	-0.52	97.49	-3.21	2089.12	-3.19
	(174.89,279.31)	(-1.26,-0.98)	(1897.29,2377)	(-0.63,-0.42)	(90.28,102)	(-3.48,-2.94)	(1952.72,2175.06)	(-3.46,-2.91)
**Mexico**	329.42	-0.3	2797.04	-0.08	113.21	-0.1	2125.69	-0.03
	(261.01,403.11)	(-0.37,-0.24)	(2384.64,3344.04)	(-0.12,-0.03)	(100.89,125.2)	(-0.36,0.17)	(1892.12,2373.05)	(-0.29,0.22)
**Micronesia**	394.33	0.1	2850.1	0.07	266.43	0.03	6279.72	0
**(Federated States of)**	(309.18,495.8)	(0.06,0.13)	(2550.23,3189.9)	(0.05,0.08)	(213.65,330.4)	(0.01,0.05)	(4925.69,8031.96)	(-0.03,0.04)
**Monaco**	155.29	-0.62	1454.73	-0.41	49.33	-2.48	900.15	-2.59
	(122.39,196.46)	(-0.68,-0.55)	(1279.26,1647.73)	(-0.46,-0.35)	(40.1,59.33)	(-2.56,-2.4)	(731.12,1090.66)	(-2.67,-2.51)
**Mongolia**	669.06	-0.9	4213.77	-0.2	219.59	-1.73	3892.31	-1.89
	(547.48,799.77)	(-1.09,-0.7)	(3806.63,4658.65)	(-0.23,-0.16)	(190.33,247.4)	(-1.98,-1.48)	(3366.09,4387.1)	(-2.17,-1.6)
**Montenegro**	364.68	0.22	3731.24	0.13	212.83	0.97	3538.19	0.29
	(288.85,453.3)	(0.17,0.27)	(3294.65,4185.02)	(0.09,0.17)	(186.68,239.2)	(0.73,1.2)	(3127.13,3990.74)	(0.17,0.42)
**Morocco**	995.04	0	6850.37	0.04	267.15	-0.35	5211.8	-0.69
	(874.59,1143.15)	(-0.06,0.05)	(6335.79,7416.41)	(0.01,0.06)	(210.84,310.86)	(-0.4,-0.3)	(3980.8,6188.35)	(-0.73,-0.64)
**Mozambique**	326	-0.3	2348.63	0.17	49.06	1.56	1060.36	1.71
	(257.17,403.42)	(-0.39,-0.21)	(2063.78,2682.72)	(0.15,0.19)	(36.92,61.05)	(1.38,1.74)	(811.04,1321.95)	(1.5,1.93)
**Myanmar**	241.69	-0.51	1927.91	-0.18	138.21	-1.25	2773.11	-1.64
	(208.57,279.86)	(-0.59,-0.42)	(1745.9,2132.74)	(-0.22,-0.15)	(113.02,171.61)	(-1.34,-1.16)	(2241.08,3446.11)	(-1.75,-1.53)
**Namibia**	344.67	-0.24	2422.7	-0.12	104.17	-0.17	2109.1	-0.28
	(269.37,434.93)	(-0.27,-0.22)	(2165.07,2723.96)	(-0.13,-0.1)	(81.37,129.06)	(-0.46,0.11)	(1621.51,2684.92)	(-0.6,0.05)

location	Incidence		Prevalence		Death		DALY	
	ASR, per 100,000, 2021	EAPC, %, 1990-2021	ASR, per 100,000, 2021	EAPC, %, 1990-2021	ASR, per 100,000, 2021	EAPC, %, 1990-2021	ASR, per 100,000, 2021	EAPC, %, 1990-2021
**Nauru**	416.09 (325.25,526.98)	0.13 (0.11,0.14)	3148.77 (2827.14,3541.63)	-0.01 (-0.05,0.03)	432.64 (361.02,517.42)	0.13 (-0.14,0.4)	10681.95 (8619.33,13238.75)	0.09 (-0.21,0.39)
**Nepal**	511.87 (431.32,615.36)	-0.19 (-0.23,-0.15)	3740.04 (3407.51,4131.26)	-0.11 (-0.14,-0.09)	136.53 (111.67,169.87)	0.45 (0.28,0.62)	2890.07 (2320.57,3596.09)	0.11 (-0.07,0.28)
**Netherlands**	178.54 (145.74,217.28)	-2.29 (-2.49,-2.08)	1486.27 (1306.66,1698.66)	-1.42 (-1.54,-1.3)	37.42 (32.5,40.31)	-4.82 (-5.04,-4.6)	633.61 (573.46,675.33)	-5.26 (-5.47,-5.04)
**New Zealand**	257.38 (214.88,304.93)	-2.54 (-2.74,-2.35)	2442.82 (2022.81,2978.33)	-1.49 (-1.61,-1.37)	60.98 (52.78,65.35)	-3.81 (-3.89,-3.72)	1048.58 (948.57,1108.69)	-4.23 (-4.37,-4.1)
**Nicaragua**	309.06 (251.08,377.51)	-0.11 (-0.13,-0.09)	2470.7 (2208.78,2752.4)	-0.05 (-0.06,-0.04)	81.93 (70.19,95.03)	0.06 (-0.16,0.28)	1524.24 (1300.65,1784.47)	-0.19 (-0.38,-0.01)
**Niger**	350.99 (282.03,429.69)	-0.23 (-0.29,-0.18)	2253.94 (2012.56,2533.09)	0.05 (0,0.1)	72.56 (51.59,98.73)	0.24 (0.14,0.33)	1413.47 (999.77,1956.91)	0.15 (0.04,0.26)
**Nigeria**	389.43 (306.22,488.32)	0.03 (-0.01,0.06)	2745.67 (2322.04,3286.99)	0.47 (0.43,0.51)	109.48 (90.67,129.23)	0.14 (0.03,0.26)	2031.49 (1637.96,2451.09)	-0.02 (-0.14,0.11)
**Niue**	390.06 (306.84,494.72)	0.11 (0.09,0.12)	3033.36 (2724.94,3399.23)	0.15 (0.13,0.17)	243.51 (206.45,279.74)	-0.1 (-0.17,-0.03)	5269.82 (4331.65,6254.32)	-0.22 (-0.3,-0.14)
**North Macedonia**	391.57 (322.76,471.36)	-0.01 (-0.08,0.06)	3423.57 (3014.96,3835.59)	-0.13 (-0.15,-0.11)	195.86 (170.12,224.26)	-0.58 (-1.02,-0.13)	3156.91 (2698.2,3663.31)	-1.3 (-1.63,-0.96)
**Northern Mariana Islands**	394.3 (306.25,508.26)	0.22 (0.19,0.25)	3247.51 (2924.13,3635.82)	0.12 (0.08,0.16)	152.09 (134.52,164.56)	0.67 (0.49,0.85)	3338.98 (2926.76,3574.47)	0.63 (0.46,0.81)
**Norway**	169.91 (133.41,214.59)	-1.98 (-2.16,-1.79)	1478.62 (1245.13,1762.63)	-1.04 (-1.16,-0.92)	40.88 (35.33,43.73)	-4.94 (-5.12,-4.77)	698.31 (633.77,740.7)	-5.28 (-5.39,-5.17)
**Oman**	1027.08 (829.69,1253.84)	-0.15 (-0.27,-0.04)	6854.91 (6258.97,7535.67)	0.3 (0.23,0.38)	179.25 (150.33,211.7)	-1.4 (-1.53,-1.27)	3393.69 (2837.02,4065.11)	-1.87 (-2.05,-1.7)
**Pakistan**	673.64 (540.86,825.31)	0.15 (0.05,0.26)	5155.28 (4349.29,6133.65)	0.15 (0.12,0.17)	183.45 (154.6,226.81)	0.81 (0.69,0.94)	4069.01 (3417.01,4985.77)	0.72 (0.57,0.87)
**Palau**	388.02 (305.58,500.84)	0.41 (0.38,0.45)	3050.32 (2730.34,3393.14)	0.44 (0.4,0.48)	233.62 (197.27,272.14)	-0.02 (-0.11,0.07)	5232.97 (4330.71,6240.48)	-0.12 (-0.2,-0.03)
**Palestine**	942.08 (762.36,1163.09)	-0.15 (-0.19,-0.12)	6302.59 (5748.27,6947.22)	0 (-0.03,0.02)	188.68 (162.69,211.7)	-1.51 (-1.77,-1.25)	3458.69 (3014.4,3871.78)	-1.66 (-1.88,-1.44)
**Panama**	298.49	-0.18	2643.61	0.16	54.48	-1.97	1097.33	-1.8

location	Incidence		Prevalence		Death		DALY	
	ASR, per 100,000, 2021	EAPC, %, 1990-2021	ASR, per 100,000, 2021	EAPC, %, 1990-2021	ASR, per 100,000, 2021	EAPC, %, 1990-2021	ASR, per 100,000, 2021	EAPC, %, 1990-2021
	(234.77,372.79)	(-0.21,-0.15)	(2361.03,2949.29)	(0.15,0.16)	(43.03,64.93)	(-2.21,-1.72)	(870.2,1306.77)	(-2.06,-1.54)
**Papua New Guinea**	356.49	-0.02	2752.72	0.14	144.45	0.26	3369.29	0.14
	(280.14,441.74)	(-0.06,0.01)	(2465.15,3062.85)	(0.12,0.17)	(109.76,186.31)	(0.14,0.37)	(2518.53,4367.45)	(0.03,0.25)
**Paraguay**	178.3	-0.15	2027.55	0.03	86.53	-0.43	1786.15	-0.48
	(142.09,218.31)	(-0.17,-0.12)	(1770.76,2311.65)	(0.01,0.05)	(67.26,106.48)	(-0.67,-0.19)	(1391.01,2223.94)	(-0.67,-0.29)
**Peru**	222.24	-0.47	2233.02	0.4	44.8	-2.25	916.07	-2.16
	(178.43,271.49)	(-0.58,-0.36)	(1984.49,2504.74)	(0.32,0.48)	(34.86,55.87)	(-2.73,-1.77)	(723.47,1141.69)	(-2.65,-1.66)
**Philippines**	177.16	-0.67	1881.91	-0.4	150.42	-0.35	3326.47	-0.16
	(140.1,219.91)	(-0.92,-0.43)	(1604.13,2205.58)	(-0.57,-0.23)	(129.19,171.93)	(-0.43,-0.28)	(2831.63,3850.53)	(-0.22,-0.1)
**Poland**	227.31	-2.98	2904.45	-0.89	113.26	-3.08	1991.47	-3.53
	(195.03,261.71)	(-3.26,-2.69)	(2469.01,3423.45)	(-0.99,-0.79)	(100.87,122.58)	(-3.22,-2.94)	(1811.44,2148.09)	(-3.7,-3.36)
**Portugal**	72.93	-3.44	947.1	-1.27	36.8	-4.22	720.75	-3.96
	(61.36,85.88)	(-3.78,-3.11)	(806.24,1099.61)	(-1.41,-1.13)	(32.35,39.41)	(-4.47,-3.98)	(663.52,762.18)	(-4.25,-3.67)
**Puerto Rico**	351.5	-0.56	3456.2	0.05	53.62	-3.84	1139.29	-3.51
	(284.69,430.43)	(-0.65,-0.47)	(3089.08,3862.02)	(0.04,0.06)	(44.64,61.73)	(-4.07,-3.61)	(955.18,1324.13)	(-3.76,-3.25)
**Qatar**	1032.07	-0.26	7143.84	-0.17	123.16	-4.51	2139.49	-4.5
	(800.61,1299.53)	(-0.34,-0.19)	(6501.02,7864.41)	(-0.21,-0.12)	(97.95,147.94)	(-5.27,-3.75)	(1690.41,2627.18)	(-5.17,-3.83)
**Republic of Korea**	96.28	-2.18	841.83	-1.12	28.27	-3.03	470.99	-3.26
	(79.26,116.32)	(-2.57,-1.79)	(748.12,948.82)	(-1.24,-0.99)	(22.87,32.57)	(-3.3,-2.75)	(402.59,526.79)	(-3.51,-3.01)
**Republic of Moldova**	511.83	-0.9	3389.82	-0.04	230.52	-2.52	4379.75	-2.05
	(444.58,586.7)	(-1.02,-0.79)	(3077.65,3744.47)	(-0.06,-0.03)	(209.69,252.49)	(-2.83,-2.21)	(4010.89,4794.82)	(-2.39,-1.71)
**Romania**	364.29	-1.75	3277.42	-0.9	154.19	-2.45	2837.96	-2.45
	(322.16,417.78)	(-2.09,-1.42)	(2918.82,3661.68)	(-1.07,-0.73)	(140.01,169.13)	(-2.63,-2.27)	(2575.38,3113.27)	(-2.68,-2.22)
**Russian Federation**	727.97	-0.35	5279.14	0.23	212.91	-1.61	4082.19	-1.75
	(586.2,880.94)	(-0.65 -0.04)	(4568.46,6189.43)	(0.12,0.34)	(195.61,229.02)	(-2.1,-1.12)	(3760.5,4389.11)	(-2.32,-1.18)
**Rwanda**	294.24	-0.37	1986.34	0.01	65.09	-2.22	1306.89	-2.69
	(231.49,366.29)	(-0.43,-0.3)	(1756.9,2254.25)	(-0.04,0.06)	(46.31,88.1)	(-2.59,-1.86)	(923.91,1754.34)	(-3.08,-2.28)
**Saint Kitts and Nevis**	387.91	-0.09	3262.02	0.06	91.15	-2.76	1718.25	-3.05
	(303.07,485.65)	(-0.13,-0.05)	(2934.98,3622.48)	(0.04,0.09)	(78.72,103.5)	(-2.99,-2.54)	(1451.68,2018.72)	(-3.29,-2.81)
**Saint Lucia**	390.38	-0.06	3359.6	0.1	48.5	-4.25	922.07	-3.83
	(309.39,486.87)	(-0.1,-0.01)	(3004.06,3761.81)	(0.07,0.12)	(40.2,56.66)	(-4.67,-3.83)	(770.21,1080.92)	(-4.2,-3.47)

location	Incidence		Prevalence		Death		DALY	
	ASR, per 100,000, 2021	EAPC, %, 1990-2021	ASR, per 100,000, 2021	EAPC, %, 1990-2021	ASR, per 100,000, 2021	EAPC, %, 1990-2021	ASR, per 100,000, 2021	EAPC, %, 1990-2021
**Saint Vincent and the Grenadines**	372.87 (293.7,464.65)	0.04 (0,0.07)	3120.2 (2793.14,3482.11)	0.22 (0.21,0.23)	100.97 (90.71,111.62)	-2.03 (-2.28,-1.79)	1822.04 (1633.37,2020.96)	-2.2 (-2.41,-1.99)
**Samoa**	410.13 (319.2,524.12)	0.06 (0.05,0.08)	3189.58 (2865.16,3564.88)	0.11 (0.09,0.12)	226.72 (194.95,270.93)	0.37 (0.33,0.41)	4993.89 (4206.29,6087.24)	0.43 (0.39,0.48)
**San Marino**	154.38 (120.56,195.36)	-0.57 (-0.65,-0.49)	1434.39 (1259.89,1625)	-0.37 (-0.42,-0.32)	23.19 (15.63,32)	-3.28 (-3.64,-2.92)	429.12 (298.92,591.84)	-3.14 (-3.44,-2.83)
**Sao Tome and Principe**	389.79 (304.27,497.04)	0.09 (0.06,0.12)	2709.64 (2411.03,3048.19)	0.34 (0.32,0.37)	103.54 (89.77,120.61)	0.99 (0.86,1.12)	2006.42 (1715.18,2352.64)	0.85 (0.68,1.03)
**Saudi Arabia**	920.14 (777.97,1085.34)	0.16 (0.04,0.28)	7341.64 (6747.53,7966.8)	0.59 (0.54,0.64)	185.9 (158.13,219.08)	-0.72 (-0.91,-0.52)	4219.77 (3530.63,5082.93)	-0.42 (-0.64,-0.21)
**Senegal**	390.43 (314.42,472.12)	-0.28 (-0.33,-0.24)	2828.37 (2519.16,3205.75)	0.03 (0,0.07)	121.62 (95.49,151.29)	-0.39 (-0.49,-0.3)	2346.78 (1836.03,2939.94)	-0.52 (-0.62,-0.42)
**Serbia**	468.45 (404.2,544.85)	-0.6 (-0.82,-0.38)	3958.92 (3508.6,4363.42)	-0.43 (-0.53,-0.33)	174.28 (150.25,200.22)	-2.56 (-2.84,-2.27)	2952.02 (2558.27,3364.12)	-2.43 (-2.68,-2.19)
**Seychelles**	223.44 (175.39,287.93)	0.08 (0.05,0.1)	2073.95 (1842.02,2344.23)	0.15 (0.13,0.17)	87.6 (78.75,97.97)	-1.27 (-1.4,-1.15)	1807.8 (1629.13,2020.44)	-1.57 (-1.7,-1.45)
**Sierra Leone**	393.12 (315.9,485.57)	-0.29 (-0.31,-0.27)	2614.24 (2340.19,2943.79)	-0.12 (-0.17,-0.08)	129.53 (101.84,159.08)	0.2 (0.01,0.39)	2597.78 (1984.64,3281.59)	0.24 (0.04,0.45)
**Singapore**	149.87 (117.59,187.95)	-0.63 (-0.83,-0.43)	1350.84 (1280.81,1431.76)	-0.29 (-0.38,-0.2)	46.74 (41.78,49.85)	-3.96 (-4.2,-3.72)	934.09 (866.47,985.49)	-4.11 (-4.3,-3.91)
**Slovakia**	357.35 (308.07,412.72)	-1.09 (-1.5,-0.69)	2987.22 (2644.16,3325.7)	-0.62 (-0.83,-0.41)	184.77 (160.51,206.7)	-1.77 (-1.84,-1.7)	3080.71 (2709.5,3443.39)	-2.33 (-2.42,-2.24)
**Slovenia**	286.63 (226.17,359.69)	-1.33 (-2.14,-0.51)	3247.42 (2846.45,3641.34)	-0.58 (-0.87,-0.29)	45.95 (39.17,51.61)	-3.85 (-4.04,-3.65)	822.83 (715.65,920.55)	-4.05 (-4.23,-3.86)
**Solomon Islands**	426.82 (330.7,533.32)	0.31 (0.23,0.4)	3043.63 (2737.79,3403.5)	0.21 (0.19,0.24)	275.17 (228.47,339.14)	-0.2 (-0.32,-0.08)	6228.54 (5006.15,7901.08)	-0.2 (-0.34,-0.06)
**Somalia**	337.69 (266.5,416.64)	-0.16 (-0.21,-0.12)	2063.39 (1815.08,2356.54)	-0.02 (-0.04,0.01)	68.48 (48.16,95.21)	0.2 (0.07,0.34)	1549.45 (1070.27,2167.12)	0.1 (-0.03,0.23)
**South Africa**	380.13 (300.49,471.61)	-0.52 (-0.61,-0.43)	2839.86 (2396.41,3389.41)	-0.31 (-0.38,-0.24)	78.04 (71.05,84.66)	0 (-0.39,0.39)	1568.46 (1435.5,1705.97)	-0.28 (-0.67,0.11)
**South Sudan**	327.21	0.1	2300.26	0.12	81.22	0.27	1783.57	0.23

location	Incidence		Prevalence		Death		DALY	
	ASR, per 100,000, 2021	EAPC, %, 1990-2021	ASR, per 100,000, 2021	EAPC, %, 1990-2021	ASR, per 100,000, 2021	EAPC, %, 1990-2021	ASR, per 100,000, 2021	EAPC, %, 1990-2021
	(259.95,413.78)	(0.06,0.13)	(2025.93,2607.96)	(0.07,0.17)	(61.25,106.99)	(0.2,0.34)	(1308.71,2389.28)	(0.12,0.33)
**Spain**	143.48 (122.62,167.12)	-1.48 (-1.7,-1.25)	1266.34 (1127.84,1404.8)	-0.47 (-0.53,-0.41)	35.37 (30.69,38.04)	-3.48 (-3.6,-3.35)	678.71 (620.16,718.29)	-3.4 (-3.52,-3.28)
**Sri Lanka**	248.5 (210.55,290.2)	-0.31 (-0.43,-0.19)	2204.12 (1990.83,2458.31)	-0.07 (-0.14,0)	94.59 (64.82,125.44)	-0.81 (-1.02,-0.59)	1963.57 (1330.01,2662.39)	-0.96 (-1.18,-0.74)
**Sudan**	973.5 (845.4,1130.98)	-0.39 (-0.43,-0.34)	6817.59 (6290.96,7433.74)	0.05 (0.03,0.06)	255.88 (202.4,330.73)	-1.08 (-1.14,-1.02)	5338.32 (4083.7,7066.87)	-1.3 (-1.36,-1.24)
**Suriname**	404.09 (321.1,502.45)	-0.03 (-0.06,-0.01)	3471.68 (3126.82,3886.67)	0.15 (0.13,0.17)	90.94 (69.95,113.45)	-1.91 (-2.19,-1.63)	2036.82 (1594.71,2533.57)	-1.88 (-2.17,-1.6)
**Sweden**	226.81 (177.47,284.75)	-1.48 (-1.75,-1.22)	1459.82 (1245.57,1714.73)	-0.44 (-0.51,-0.36)	51.8 (44.14,57.63)	-4 (-4.1,-3.9)	860.82 (751.7,958.42)	-4.15 (-4.21,-4.09)
**Switzerland**	149.87 (122.63,182.47)	-1.4 (-1.75,-1.05)	1249.13 (1092.19,1416.02)	-0.81 (-0.96,-0.66)	39.96 (33.2,43.53)	-4.24 (-4.33,-4.15)	639.19 (566.36,688.5)	-4.57 (-4.63,-4.51)
**Syrian Arab Republic**	1130.3 (993.53,1305.6)	0.05 (0.01,0.1)	6859.54 (6321.62,7467.57)	0.07 (0.05,0.09)	353.01 (281.64,432.02)	-0.47 (-0.58,-0.36)	6688.8 (5230.44,8518.87)	-0.87 (-1.01,-0.74)
**Taiwan (Province of China)**	303.19 (243.88,375.54)	-0.72 (-0.82,-0.62)	2817.32 (2556.51,3144.34)	-0.18 (-0.2,-0.16)	33.61 (30,36.21)	-2.46 (-2.71,-2.21)	684.68 (626.58,728.31)	-2.04 (-2.28,-1.8)
**Tajikistan**	774.65 (678.93,883.79)	1.09 (1.01,1.18)	4105.81 (3730.25,4525.27)	0.26 (0.21,0.31)	244.92 (206.56,281.62)	-0.97 (-1.31,-0.63)	4400.38 (3740.14,5106.84)	-1.3 (-1.61,-0.99)
**Thailand**	169.58 (139.96,200.89)	-1.14 (-1.26,-1.03)	1732.21 (1551.16,1933.28)	-0.29 (-0.33,-0.25)	47.05 (36.8,58.63)	-2.54 (-2.8,-2.27)	1039.67 (825.63,1278.93)	-2.23 (-2.5,-1.95)
**Timor-Leste**	221.76 (174.75,277.01)	0.37 (0.28,0.45)	1887.26 (1692.82,2134.91)	0.31 (0.28,0.34)	156.95 (121.11,195.97)	1.12 (0.93,1.32)	3162.99 (2433.53,4007.89)	0.92 (0.69,1.15)
**Togo**	386.94 (305.72,475.8)	-0.13 (-0.17,-0.08)	2502.85 (2229.98,2825.33)	0.03 (0,0.05)	117 (89.49,149.18)	-0.17 (-0.38,0.04)	2348.16 (1747.13,3034.4)	-0.18 (-0.41,0.04)
**Tokelau**	373.08 (292.48,478.98)	0.43 (0.42,0.44)	2845.41 (2560.17,3184.56)	0.49 (0.47,0.51)	205.41 (164.75,248.55)	-0.31 (-0.39,-0.24)	4491.08 (3546.59,5579.27)	-0.34 (-0.41,-0.27)
**Tonga**	403.04 (315.34,515.14)	0.14 (0.12,0.15)	3153.6 (2832.02,3513.92)	0.14 (0.13,0.15)	139.13 (113.6,166.57)	0.15 (0.03,0.28)	3065.33 (2467.28,3781.66)	0.08 (-0.03,0.19)
**Trinidad and Tobago**	422.86 (336.91,521.43)	-0.39 (-0.43,-0.34)	3917.34 (3495.62,4379.84)	0.11 (0.1,0.13)	105.96 (83.2,131.89)	-3.12 (-3.37,-2.86)	2270.93 (1754.62,2870.76)	-2.98 (-3.26,-2.69)

location	Incidence		Prevalence		Death		DALY	
	ASR, per 100,000, 2021	EAPC, %, 1990-2021	ASR, per 100,000, 2021	EAPC, %, 1990-2021	ASR, per 100,000, 2021	EAPC, %, 1990-2021	ASR, per 100,000, 2021	EAPC, %, 1990-2021
**Tunisia**	847.53 (711,1026.1)	-0.24 (-0.3,-0.18)	6011.91 (5516.8,6558.82)	-0.01 (-0.03,0.02)	163.59 (121.84,215.67)	-1.24 (-1.37,-1.12)	3036.73 (2267.13,4065.15)	-1.35 (-1.48,-1.23)
**Turkey**	665.07 (568.25,784.58)	-0.75 (-0.86,-0.64)	5365.86 (4895.52,5874.65)	-0.55 (-0.6,-0.51)	133.38 (109.89,155.94)	-1.29 (-1.59,-0.98)	2419.28 (2008.07,2856.99)	-1.92 (-2.15,-1.68)
**Turkmenistan**	689.66 (588.79,796.61)	-0.44 (-0.57,-0.31)	4460.74 (4029.37,4943.29)	0.21 (0.19,0.22)	343.68 (280.94,420)	-1.5 (-1.9,-1.11)	6512.7 (5219.52,8027.65)	-1.57 (-1.99,-1.15)
**Tuvalu**	385.73 (301.45,495.08)	0.3 (0.29,0.31)	2817.02 (2535.6,3142.5)	0.34 (0.31,0.36)	269.54 (234.98,314.27)	0.01 (-0.04,0.06)	6200.06 (5251.54,7307.55)	-0.01 (-0.06,0.03)
**Uganda**	291.89 (235.73,356.69)	-0.41 (-0.49,-0.32)	2050.67 (1811.64,2314.69)	-0.11 (-0.16,-0.06)	71.03 (56.38,91.34)	-0.61 (-1,-0.23)	1490 (1191.54,1887.39)	-0.71 (-1.12,-0.3)
**Ukraine**	752.32 (608.03,916.09)	0.13 (-0.1,0.37)	4518.85 (3867.46,5313.07)	0.12 (0.08,0.16)	373.47 (291.58,459.07)	-0.3 (-0.82,0.23)	6522.69 (5003.4,8125.67)	-0.3 (-0.83,0.24)
**United Arab Emirates**	1084.31 (843.39,1343.89)	-0.11 (-0.15,-0.07)	7608.89 (6995.21,8286.01)	0.12 (0.07,0.16)	167.71 (136.64,196.33)	0.13 (-0.41,0.67)	2923.67 (2395.62,3422)	-0.86 (-1.29,-0.43)
**United Kingdom**	141.62 (115.22,170.01)	-2.12 (-2.48,-1.77)	1363.38 (1162.34,1599.93)	-1.45 (-1.62,-1.27)	52.11 (46.86,54.65)	-4.67 (-4.85,-4.49)	983.93 (921.73,1021.77)	-4.76 (-4.96,-4.56)
**United Republic of Tanzania**	321.55 (264.22,386.47)	0.24 (0.2,0.29)	2363.96 (2095.97,2669.73)	0.67 (0.61,0.73)	96.28 (72.01,123.39)	1.04 (0.92,1.17)	2051.06 (1526.6,2613.31)	0.92 (0.8,1.05)
**United States of America**	170.36 (143.9,197.67)	-3.66 (-3.94,-3.38)	1488.78 (1263.92,1756.36)	-2.57 (-2.75,-2.38)	78.92 (69.93,83.85)	-3.01 (-3.15,-2.86)	1527.33 (1412.25,1595.15)	-2.9 (-3.05,-2.75)
**United States Virgin Islands**	384.68 (303.56,482.22)	0.28 (0.23,0.33)	3369.91 (3024.74,3757.1)	0.4 (0.37,0.43)	102.62 (83.61,125.67)	-2.25 (-2.39,-2.11)	1953.39 (1594.1,2390.09)	-2.25 (-2.39,-2.1)
**Uruguay**	204.18 (159.75,254.81)	-0.96 (-1.1,-0.82)	1485.26 (1318.8,1682.6)	-0.39 (-0.47,-0.31)	60.55 (55.5,63.77)	-3.04 (-3.23,-2.85)	1196.72 (1130.23,1250.88)	-2.99 (-3.17,-2.8)
**Uzbekistan**	1206.01 (1125.02,1305.45)	2.52 (2.15,2.9)	5015.59 (4660.91,5391.59)	1 (0.89,1.11)	339.48 (295.64,382.01)	0.3 (-0.05,0.65)	6218.8 (5449.76,7058.07)	0.05 (-0.31,0.42)
**Vanuatu**	456.38 (354.88,574.23)	0.24 (0.21,0.27)	3392.41 (3036.9,3799.35)	0.16 (0.15,0.18)	308.39 (260.86,357.06)	-0.1 (-0.14,-0.05)	7189.7 (6037.23,8411.26)	-0.14 (-0.2,-0.08)
**Venezuela (Bolivarian Republic of)**	323.97 (279.3,375.28)	-0.56 (-0.65,-0.48)	2688.96 (2428.9,2974.7)	-0.32 (-0.34,-0.29)	141.4 (112.21,177.15)	-0.98 (-1.26,-0.7)	2941.77 (2286.73,3718.26)	-1.08 (-1.35,-0.81)
**Viet Nam**	232.72	0.38	1961.27	0.49	76.99	0.96	1464.59	0.86

location	Incidence		Prevalence		Death		DALY	
	ASR, per 100,000, 2021	EAPC, %, 1990-2021	ASR, per 100,000, 2021	EAPC, %, 1990-2021	ASR, per 100,000, 2021	EAPC, %, 1990-2021	ASR, per 100,000, 2021	EAPC, %, 1990-2021
	(201.98,270.45)	(0.3,0.46)	(1779.89,2182.1)	(0.43,0.54)	(63.45,90.68)	(0.77,1.16)	(1198.7,1740.5)	(0.64,1.09)
**Yemen**	948.5 (819.7,1114.36)	-0.31 (-0.36,-0.25)	6182.82 (5696.13,6755.92)	-0.06 (-0.07,-0.05)	263.54 (201.84,344.23)	-0.85 (-0.94,-0.75)	5442.56 (4100.16,7104.85)	-1.11 (-1.21,-1.01)
**Zambia**	305.26 (242.23,377.4)	-0.36 (-0.43,-0.29)	2109.97 (1848.31,2384.45)	-0.01 (-0.05,0.03)	92.7 (71.66,117.34)	1.29 (1.18,1.4)	1937.07 (1461.43,2469.87)	1.13 (1,1.26)
**Zimbabwe**	389.53 (313.51,469.31)	0.39 (0.31,0.47)	2555.2 (2267.36,2867.52)	0.15 (0.08,0.21)	118.54 (97.5,145.32)	1.75 (1.21,2.29)	2404.18 (1938.12,3035.67)	2.02 (1.42,2.61)

Table S5 Frontier DALYs, and effective difference by country or territory.

Location	SDI	Age standardized DALYs	Frontier DALYs	Effective difference	Effective difference rank (Age standardized DALYs rank)
**Afghanistan**	0.34	6178.27 (4747.55 - 7813.31)	737.16	5441.11	192 (192)
**Albania**	0.71	2558.27 (2184.88 - 2998.29)	733.05	1825.21	128 (128)
**Algeria**	0.66	3451.99 (2806.95 - 4159.87)	730.66	2721.33	162 (164)
**American Samoa**	0.72	4053.34 (3456.83 - 4752.36)	730.32	3323.02	173 (173)
**Andorra**	0.87	724.42 (539.1 - 929.5)	527.44	196.99	18 (14)
**Angola**	0.45	2529.04 (1954.37 - 3169.14)	737.19	1791.85	127 (127)
**Antigua and Barbuda**	0.75	1306.35 (1226.11 - 1441.33)	726.21	580.13	46 (46)
**Argentina**	0.72	1179.87 (1113.64 - 1234.14)	730.24	449.62	36 (40)
**Armenia**	0.70	3788.46 (3403.75 - 4233.65)	738.62	3049.84	169 (169)
**Australia**	0.84	768.62 (696.1 - 814.23)	575.81	192.81	17 (16)
**Austria**	0.85	1121.64 (1017.81 - 1189.7)	574.07	547.57	43 (35)
**Azerbaijan**	0.69	5496.28 (4818.68 - 6182.32)	733.65	4762.62	190 (190)
**Bahamas**	0.81	1624.95 (1339.72 - 1979.27)	678.52	946.44	66 (68)
**Bahrain**	0.75	2796.56 (2418.62 - 3179.47)	723.12	2073.44	137 (137)
**Bangladesh**	0.49	2366.92 (1856.38 - 2929.73)	737.55	1629.37	119 (121)
**Barbados**	0.75	1150.8 (936.24 - 1387.58)	721.72	429.07	34 (37)
**Belarus**	0.78	6244.29 (5262.84 - 7270.21)	716.33	5527.96	196 (196)
**Belgium**	0.85	698.85 (638.41 - 740.15)	580.31	118.54	12 (11)
**Belize**	0.61	1363.17 (1223.34 - 1513.59)	730.26	632.91	49 (48)
**Benin**	0.37	1514.95 (1244.52 - 1848.81)	733.98	780.96	59 (59)
**Bermuda**	0.82	1283.78 (1113.11 - 1520.67)	656.85	626.93	48 (44)
**Bhutan**	0.47	2299.54 (1757.99 - 2871.27)	732.99	1566.56	115 (116)
**Bolivia (Plurinational State of)**	0.60	1602.36 (1154.23 - 2291.24)	732.36	870	65 (67)
**Bosnia and Herzegovina**	0.72	2450.2 (2019.97 - 2859.4)	732.6	1717.6	125 (125)
**Botswana**	0.64	1574.31 (1216.62 - 1984.6)	735.07	839.25	64 (66)
**Brazil**	0.65	1469.72 (1380.14 - 1530.27)	737.72	732	54 (55)
**Brunei Darussalam**	0.81	1726.32 (1525.62 - 1929.94)	665.43	1060.89	76 (71)
**Bulgaria**	0.77	3852.25 (3333.94 - 4409.66)	697.99	3154.26	171 (170)
**Burkina Faso**	0.29	1884.27 (1448.29 - 2432.51)	734.25	1150.02	82 (82)

Location	SDI	Age standardized DALYs	Frontier DALYs	Effective difference	Effective difference rank (Age standardized DALYs rank)
**Burundi**	0.29	1841.44 (1452.49 - 2345.53)	730.15	1111.29	79 (79)
**Cabo Verde**	0.53	2236.65 (1830.13 - 2642.4)	733.86	1502.79	109 (110)
**Cambodia**	0.47	2251.18 (1767.77 - 2790.06)	730.33	1520.84	110 (111)
**Cameroon**	0.48	2044.68 (1551.58 - 2747.31)	733.87	1310.82	97 (98)
**Canada**	0.87	922.52 (849.26 - 968.23)	527.14	395.37	29 (24)
**Central African Republic**	0.31	3256.9 (2364.11 - 4604.4)	731.14	2525.76	155 (155)
**Chad**	0.24	2128.18 (1619.46 - 2717.98)	731.45	1396.74	104 (106)
**Chile**	0.77	808.36 (760.79 - 847.46)	710.71	97.66	11 (18)
**China**	0.72	1856.51 (1548.73 - 2159.82)	736.52	1119.98	80 (80)
**Colombia**	0.66	1502.23 (1264.42 - 1754.08)	731.14	771.09	58 (58)
**Comoros**	0.48	1526.07 (1137.18 - 1995.75)	731.18	794.89	62 (61)
**Congo**	0.58	3210.45 (2515.58 - 3995.7)	733.72	2476.73	154 (154)
**Cook Islands**	0.78	2344.82 (1931.57 - 2837.91)	700.08	1644.74	120 (118)
**Costa Rica**	0.70	1157.79 (1033.42 - 1276.35)	733.87	423.91	33 (38)
**Coted'Ivoire**	0.43	2390.53 (1895.8 - 3151.33)	735.05	1655.48	121 (122)
**Croatia**	0.80	2099.58 (1856.5 - 2326.01)	687.11	1412.47	105 (101)
**Cuba**	0.67	2017.01 (1763.5 - 2255.67)	729.16	1287.85	93 (95)
**Cyprus**	0.84	1394.96 (1225.32 - 1564.95)	605.42	789.55	60 (49)
**Czechia**	0.83	2101.42 (1857.11 - 2327.15)	650.32	1451.1	106 (102)
**Democratic People's Republic of Korea**	0.57	2594.23 (2058.22 - 3211.81)	729.42	1864.81	130 (130)
**Democratic Republic of the Congo**	0.38	2290.38 (1696.29 - 3056.52)	729.82	1560.56	114 (114)
**Denmark**	0.90	710.23 (653.57 - 756)	491.69	218.54	21 (12)
**Djibouti**	0.49	1927.16 (1417.07 - 2555.52)	731.72	1195.45	85 (85)
**Dominica**	0.75	1738.9 (1513.77 - 2064.5)	725.81	1013.09	73 (74)
**Dominican Republic**	0.62	3104.02 (2494.68 - 3824.78)	737.81	2366.21	149 (150)
**Ecuador**	0.66	1433.67 (1158.9 - 1770.44)	730.01	703.66	50 (51)
**Egypt**	0.61	6924.84 (5844.94 - 8119.21)	735.65	6189.18	202 (202)
**El Salvador**	0.56	1915.93 (1577.46 - 2311.23)	734.79	1181.13	83 (83)
**Equa-rial Guinea**	0.66	2751.44 (1907.88 - 3773.97)	733.76	2017.68	133 (133)

Location	SDI	Age standardized DALYs	Frontier DALYs	Effective difference	Effective difference rank (Age standardized DALYs rank)
**Eritrea**	0.40	1974.98 (1470.4 - 2570.23)	730.17	1244.81	91 (92)
**Es-nia**	0.84	1547.87 (1370.4 - 1716.96)	570.11	977.76	68 (63)
**Eswatini**	0.59	2334.11 (1612.79 - 3342.38)	736.01	1598.1	116 (117)
**Ethiopia**	0.36	1177.38 (959.27 - 1410.32)	732.72	444.67	35 (39)
**Fiji**	0.68	5965.61 (4721.09 - 7391.81)	731.53	5234.08	191 (191)
**Finland**	0.86	1299.12 (1163.23 - 1383.11)	578.45	720.67	52 (45)
**France**	0.84	555.98 (503.45 - 597.26)	555.98	0	3 (4)
**Gabon**	0.63	2479.16 (1901.2 - 3118.09)	737.82	1741.34	126 (126)
**Gambia**	0.41	2771.5 (2116.36 - 3471.6)	737.47	2034.03	134 (134)
**Georgia**	0.73	2565.24 (2301.9 - 2822.2)	735.92	1829.32	129 (129)
**Germany**	0.90	1096.63 (993.52 - 1160.04)	496.56	600.07	47 (33)
**Ghana**	0.56	1954.33 (1558.46 - 2392.96)	731.41	1222.92	89 (90)
**Greece**	0.79	1443.35 (1353.57 - 1503.27)	694.48	748.87	55 (53)
**Greenland**	0.83	1632.37 (1432.87 - 1918.7)	651.35	981.02	69 (69)
**Grenada**	0.67	1924.27 (1678.96 - 2155)	730.49	1193.78	84 (84)
**Guam**	0.80	3413.08 (3110.68 - 3756.02)	675.23	2737.84	164 (163)
**Guatemala**	0.54	1732.06 (1517.52 - 1959.37)	729.28	1002.77	71 (72)
**Guinea**	0.34	2209.38 (1690.78 - 2864.84)	732.8	1476.58	108 (109)
**Guinea-Bissau**	0.35	3270.22 (2536.54 - 4074.07)	733.71	2536.51	156 (156)
**Guyana**	0.65	3075.23 (2425.76 - 3871.77)	733.07	2342.15	147 (148)
**Haiti**	0.45	4370.88 (3271.69 - 5735.77)	738.13	3632.75	177 (177)
**Honduras**	0.51	3075.05 (2550.8 - 3764.35)	732.67	2342.38	148 (147)
**Hungary**	0.79	2780.98 (2470.6 - 3060.36)	714.11	2066.86	136 (136)
**Iceland**	0.88	990.46 (883.5 - 1083.79)	525.13	465.33	39 (28)
**India**	0.58	3400.03 (3098.47 - 3720.83)	732.72	2667.31	160 (162)
**Indonesia**	0.66	3043.08 (2527.28 - 3544.71)	733.24	2309.84	144 (145)
**Iran (Islamic Republic of)**	0.70	2731.3 (2517.27 - 2920.87)	730.19	2001.11	132 (132)
**Iraq**	0.66	4905.27 (3858.32 - 5827.68)	735.62	4169.65	182 (182)
**Ireland**	0.87	924.25 (829.87 - 991.72)	525.19	399.06	31 (25)

Location	SDI	Age standardized DALYs	Frontier DALYs	Effective difference	Effective difference rank (Age standardized DALYs rank)
**Israel**	0.81	599.57 (537.77 - 637.8)	599.57	0	3 (5)
**Italy**	0.81	747.14 (673.73 - 791.65)	691.62	55.52	9 (15)
**Jamaica**	0.68	1054.54 (837.96 - 1345.47)	736.52	318.02	25 (31)
**Japan**	0.87	502.25 (460.02 - 526.29)	502.25	0	3 (3)
**Jordan**	0.73	1947.22 (1603.41 - 2349.61)	732.16	1215.05	88 (87)
**Kazakhstan**	0.73	3970.5 (3569.75 - 4401.12)	738.46	3232.05	172 (172)
**Kenya**	0.52	1244.93 (964.97 - 1595.88)	732.77	512.16	42 (42)
**Kiribati**	0.53	4969.44 (4015.48 - 6139.26)	732.73	4236.71	183 (183)
**Kuwait**	0.85	2299.44 (1917.04 - 2762.48)	599.14	1700.3	124 (115)
**Kyrgyzstan**	0.60	4780.48 (4093.58 - 5490.56)	729.47	4051	181 (181)
**Lao People's Democratic Republic**	0.49	3667.08 (2896.8 - 4573.18)	733.09	2933.99	167 (167)
**Latvia**	0.83	2916.55 (2581.61 - 3230.55)	611.15	2305.39	143 (140)
**Lebanon**	0.74	1735.3 (1488.38 - 2027.36)	722.66	1012.64	72 (73)
**Lesotho**	0.51	1949.42 (1307.36 - 2989.62)	736.72	1212.7	87 (88)
**Liberia**	0.35	2264 (1760.67 - 2953.76)	732.17	1531.83	111 (112)
**Libya**	0.73	3765.46 (2952.85 - 4839.08)	736.27	3029.19	168 (168)
**Lithuania**	0.86	3332.3 (3000.98 - 3676.82)	574.52	2757.78	165 (158)
**Luxembourg**	0.88	789.76 (717.69 - 867.8)	519.51	270.25	23 (17)
**Madagascar**	0.40	2205.88 (1634.85 - 2810.29)	732.68	1473.2	107 (108)
**Malawi**	0.38	1869.9 (1556.4 - 2221.41)	734.56	1135.34	81 (81)
**Malaysia**	0.74	3180.36 (2984.07 - 3352.24)	731.94	2448.43	153 (153)
**Maldives**	0.65	1481.02 (1238.38 - 1759.98)	730.09	750.93	56 (56)
**Mali**	0.27	1439.78 (1125.23 - 1851.95)	731.59	708.2	51 (52)
**Malta**	0.80	1270.51 (1140.12 - 1387.85)	699.95	570.56	44 (43)
**Marshall Islands**	0.57	6518.18 (5093.82 - 8205.42)	730.69	5787.49	199 (199)
**Mauritania**	0.50	2126.35 (1570.63 - 2744.37)	735.44	1390.92	102 (105)
**Mauritius**	0.72	2089.12 (1952.72 - 2175.06)	731.12	1358	100 (100)
**Mexico**	0.66	2125.69 (1892.12 - 2373.05)	731.22	1394.47	103 (104)
**Micronesia (Federated States of)**	0.59	6279.72 (4925.69 - 8031.96)	731.64	5548.08	197 (197)

Location	SDI	Age standardized DALYs	Frontier DALYs	Effective difference	Effective difference rank (Age standardized DALYs rank)
**Monaco**	0.91	900.15 (731.12 - 1090.66)	443.21	456.94	37 (21)
**Mongolia**	0.62	3892.31 (3366.09 - 4387.1)	738.78	3153.53	170 (171)
**Montenegro**	0.80	3538.19 (3127.13 - 3990.74)	688.61	2849.58	166 (166)
**Morocco**	0.56	5211.8 (3980.8 - 6188.35)	731.92	4479.88	185 (185)
**Mozambique**	0.33	1060.36 (811.04 - 1321.95)	731.89	328.47	26 (32)
**Myanmar**	0.53	2773.11 (2241.08 - 3446.11)	730.19	2042.92	135 (135)
**Namibia**	0.62	2109.1 (1621.51 - 2684.92)	731.23	1377.86	101 (103)
**Nauru**	0.63	10681.95 (8619.33 - 13238.75)	735.12	9946.83	204 (204)
**Nepal**	0.43	2890.07 (2320.57 - 3596.09)	733.28	2156.79	139 (139)
**Netherlands**	0.89	633.61 (573.46 - 675.33)	493.22	140.38	13 (6)
**New Zealand**	0.85	1048.58 (948.57 - 1108.69)	574.69	473.89	40 (30)
**Nicaragua**	0.52	1524.24 (1300.65 - 1784.47)	732.17	792.08	61 (60)
**Niger**	0.17	1413.47 (999.77 - 1956.91)	1320.73	92.74	10 (50)
**Nigeria**	0.50	2031.49 (1637.96 - 2451.09)	731.81	1299.68	96 (96)
**Niue**	0.73	5269.82 (4331.65 - 6254.32)	728.96	4540.86	187 (187)
**North Macedonia**	0.75	3156.91 (2698.2 - 3663.31)	726.95	2429.96	151 (151)
**Northern Mariana Islands**	0.77	3338.98 (2926.76 - 3574.47)	697.12	2641.86	159 (159)
**Norway**	0.92	698.31 (633.77 - 740.7)	484.71	213.6	20 (10)
**Oman**	0.77	3393.69 (2837.02 - 4065.11)	697.42	2696.27	161 (161)
**Pakistan**	0.50	4069.01 (3417.01 - 4985.77)	738.34	3330.68	174 (174)
**Palau**	0.75	5232.97 (4330.71 - 6240.48)	728.95	4504.03	186 (186)
**Palestine**	0.63	3458.69 (3014.4 - 3871.78)	732.73	2725.97	163 (165)
**Panama**	0.71	1097.33 (870.2 - 1306.77)	733.24	364.09	28 (34)
**Papua New Guinea**	0.42	3369.29 (2518.53 - 4367.45)	731.47	2637.82	158 (160)
**Paraguay**	0.64	1786.15 (1391.01 - 2223.94)	730.41	1055.74	75 (76)
**Peru**	0.66	916.07 (723.47 - 1141.69)	729.8	186.27	15 (22)
**Philippines**	0.65	3326.47 (2831.63 - 3850.53)	733.03	2593.44	157 (157)
**Poland**	0.81	1991.47 (1811.44 - 2148.09)	641.76	1349.71	99 (93)
**Portugal**	0.74	720.75 (663.52 - 762.18)	720.75	0	3 (13)

Location	SDI	Age standardized DALYs	Frontier DALYs	Effective difference	Effective difference rank (Age standardized DALYs rank)
**Puer- Rico**	0.83	1139.29 (955.18 - 1324.13)	651.32	487.97	41 (36)
**Qatar**	0.85	2139.49 (1690.41 - 2627.18)	600.11	1539.38	112 (107)
**Republic of Korea**	0.89	470.99 (402.59 - 526.79)	469.33	1.66	7 (2)
**Republic of Moldova**	0.73	4379.75 (4010.89 - 4794.82)	733.96	3645.79	178 (178)
**Romania**	0.77	2837.96 (2575.38 - 3113.27)	698.09	2139.87	138 (138)
**Russian Federation**	0.81	4082.19 (3760.5 - 4389.11)	651.84	3430.35	175 (175)
**Rwanda**	0.44	1306.89 (923.91 - 1754.34)	729.54	577.35	45 (47)
**Saint Kitts and Nevis**	0.75	1718.25 (1451.68 - 2018.72)	728.11	990.14	70 (70)
**Saint Lucia**	0.67	922.07 (770.21 - 1080.92)	732.65	189.42	16 (23)
**Saint Vincent and the Grenadines**	0.64	1822.04 (1633.37 - 2020.96)	739.2	1082.85	78 (78)
**Samoa**	0.59	4993.89 (4206.29 - 6087.24)	731.2	4262.68	184 (184)
**San Marino**	0.89	429.12 (298.92 - 591.84)	429.12	0	3 (1)
**Sao -me and Principe**	0.51	2006.42 (1715.18 - 2352.64)	730.1	1276.33	92 (94)
**Saudi Arabia**	0.82	4219.77 (3530.63 - 5082.93)	661.92	3557.86	176 (176)
**Senegal**	0.41	2346.78 (1836.03 - 2939.94)	729.84	1616.94	118 (119)
**Serbia**	0.79	2952.02 (2558.27 - 3364.12)	689.37	2262.65	141 (143)
**Seychelles**	0.73	1807.8 (1629.13 - 2020.44)	734	1073.8	77 (77)
**Sierra Leone**	0.36	2597.78 (1984.64 - 3281.59)	732.65	1865.12	131 (131)
**Singapore**	0.86	934.09 (866.47 - 985.49)	572.18	361.91	27 (26)
**Slovakia**	0.81	3080.71 (2709.5 - 3443.39)	668.48	2412.23	150 (149)
**Slovenia**	0.84	822.83 (715.65 - 920.55)	596.41	226.42	22 (19)
**Solomon Islands**	0.43	6228.54 (5006.15 - 7901.08)	730.51	5498.03	195 (195)
**Somalia**	0.08	1549.45 (1070.27 - 2167.12)	1511.87	37.59	8 (64)
**South Africa**	0.68	1568.46 (1435.5 - 1705.97)	732.67	835.78	63 (65)
**South Sudan**	0.28	1783.57 (1308.71 - 2389.28)	731.78	1051.79	74 (75)
**Spain**	0.77	678.71 (620.16 - 718.29)	678.44	0.27	6 (8)
**Sri Lanka**	0.70	1963.57 (1330.01 - 2662.39)	731.62	1231.94	90 (91)
**Sudan**	0.54	5338.32 (4083.7 - 7066.87)	736.21	4602.11	188 (188)
**Suriname**	0.63	2036.82 (1594.71 - 2533.57)	738.4	1298.42	95 (97)

Location	SDI	Age standardized DALYs	Frontier DALYs	Effective difference	Effective difference rank (Age standardized DALYs rank)
**Sweden**	0.89	860.82 (751.7 - 958.42)	464.08	396.74	30 (20)
**Switzerland**	0.93	639.19 (566.36 - 688.5)	438.64	200.55	19 (7)
**Syrian Arab Republic**	0.62	6688.8 (5230.44 - 8518.87)	732.02	5956.77	201 (201)
**Taiwan (Province of China)**	0.87	684.68 (626.58 - 728.31)	524.61	160.07	14 (9)
**Tajikistan**	0.54	4400.38 (3740.14 - 5106.84)	732.81	3667.57	179 (179)
**Thailand**	0.68	1039.67 (825.63 - 1278.93)	733.01	306.65	24 (29)
**Timor-Leste**	0.44	3162.99 (2433.53 - 4007.89)	730.45	2432.54	152 (152)
**-go**	0.41	2348.16 (1747.13 - 3034.4)	733.2	1614.96	117 (120)
**-kelau**	0.69	4491.08 (3546.59 - 5579.27)	730.35	3760.73	180 (180)
**-nga**	0.63	3065.33 (2467.28 - 3781.66)	732.04	2333.3	146 (146)
**Trinidad and -bago**	0.77	2270.93 (1754.62 - 2870.76)	715.98	1554.95	113 (113)
**Tunisia**	0.68	3036.73 (2267.13 - 4065.15)	733.17	2303.57	142 (144)
**Turkey**	0.71	2419.28 (2008.07 - 2856.99)	737.8	1681.48	123 (124)
**Turkmenistan**	0.68	6512.7 (5219.52 - 8027.65)	735.09	5777.62	198 (198)
**Tuvalu**	0.58	6200.06 (5251.54 - 7307.55)	730.5	5469.56	193 (193)
**Uganda**	0.42	1490 (1191.54 - 1887.39)	732.7	757.3	57 (57)
**Ukraine**	0.76	6522.69 (5003.4 - 8125.67)	722.24	5800.44	200 (200)
**United Arab Emirates**	0.85	2923.67 (2395.62 - 3422)	604.78	2318.89	145 (141)
**United Kingdom**	0.86	983.93 (921.73 - 1021.77)	576.05	407.88	32 (27)
**United Republic of Tanzania**	0.45	2051.06 (1526.6 - 2613.31)	732.7	1318.36	98 (99)
**United States Virgin Islands**	0.82	1953.39 (1594.1 - 2390.09)	660.74	1292.65	94 (89)
**United States of America**	0.86	1527.33 (1412.25 - 1595.15)	560.57	966.76	67 (62)
**Uruguay**	0.72	1196.72 (1130.23 - 1250.88)	731.46	465.26	38 (41)
**Uzbekistan**	0.66	6218.8 (5449.76 - 7058.07)	737.3	5481.5	194 (194)
**Vanuatu**	0.47	7189.7 (6037.23 - 8411.26)	731.86	6457.85	203 (203)
**Venezuela (Bolivarian Republic of)**	0.60	2941.77 (2286.73 - 3718.26)	729.77	2212	140 (142)
**Viet Nam**	0.63	1464.59 (1198.7 - 1740.5)	735.89	728.7	53 (54)
**Yemen**	0.45	5442.56 (4100.16 - 7104.85)	733.01	4709.55	189 (189)
**Zambia**	0.51	1937.07 (1461.43 - 2469.87)	733.69	1203.38	86 (86)

Location	SDI	Age standardized DALYs	Frontier DALYs	Effective difference	Effective difference rank (Age standardized DALYs rank)
**Zimbabwe**	0.47	2404.18 (1938.12 - 3035.67)	732.54	1671.64	122 (123)

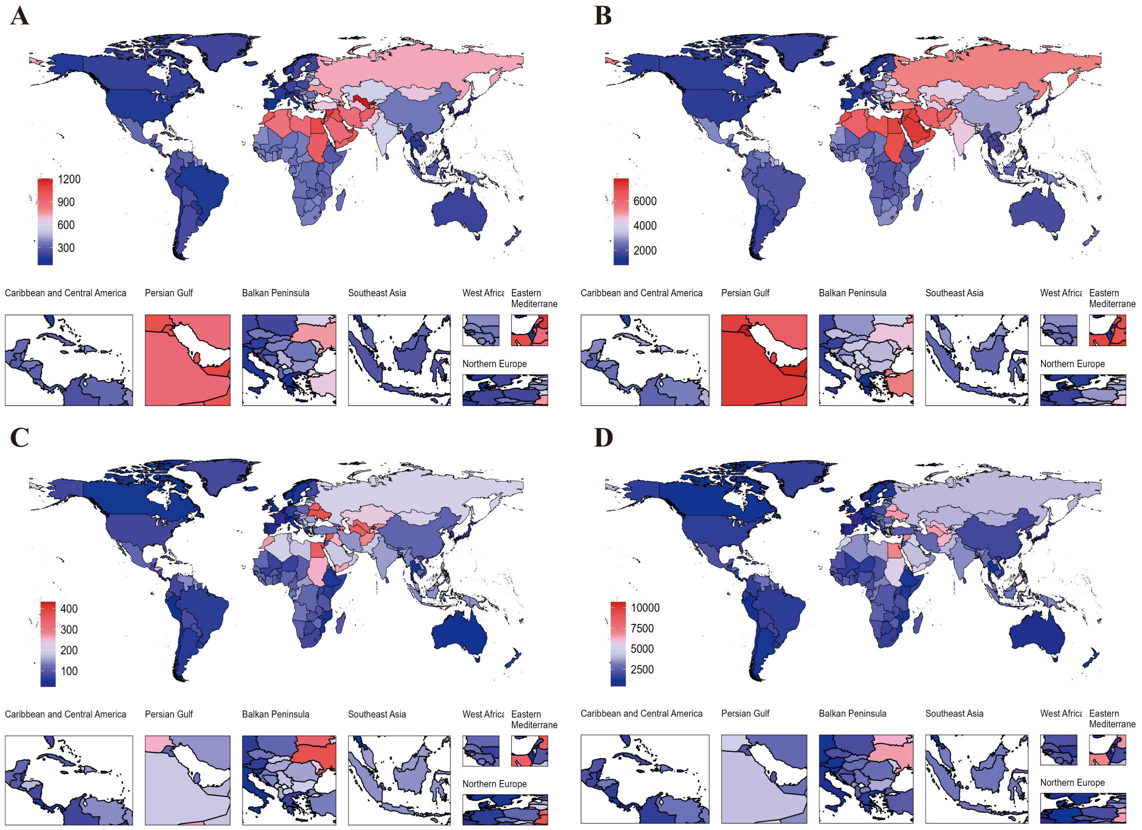

Figure S1. ASRs for IHD in both sexes across 204 countries in 2021. **Panel A.** Incidence. **Panel B.** Prevalence. **Panel C.** Death. **Panel D.** DALYs. ASR – age-standardised rate, DALY – disability-adjusted life year, IHD – ischaemic heart disease.

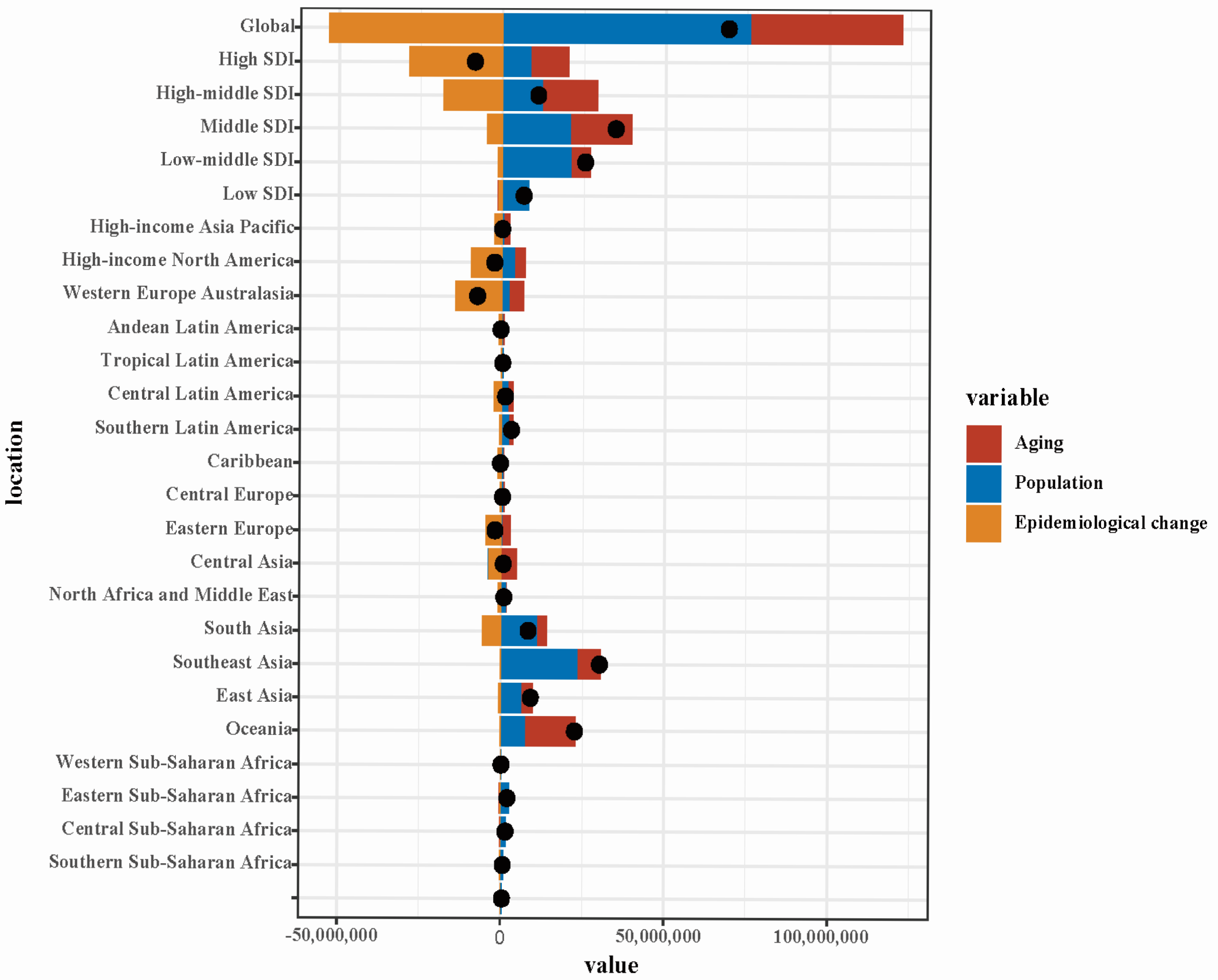

Figure S2. Global and SDI quintile-specific changes in IHD DALYs attributed to population growth, ageing, and epidemiological shifts, 1990–2021. IHD – ischaemic heart disease, SDI – sociodemographic index.

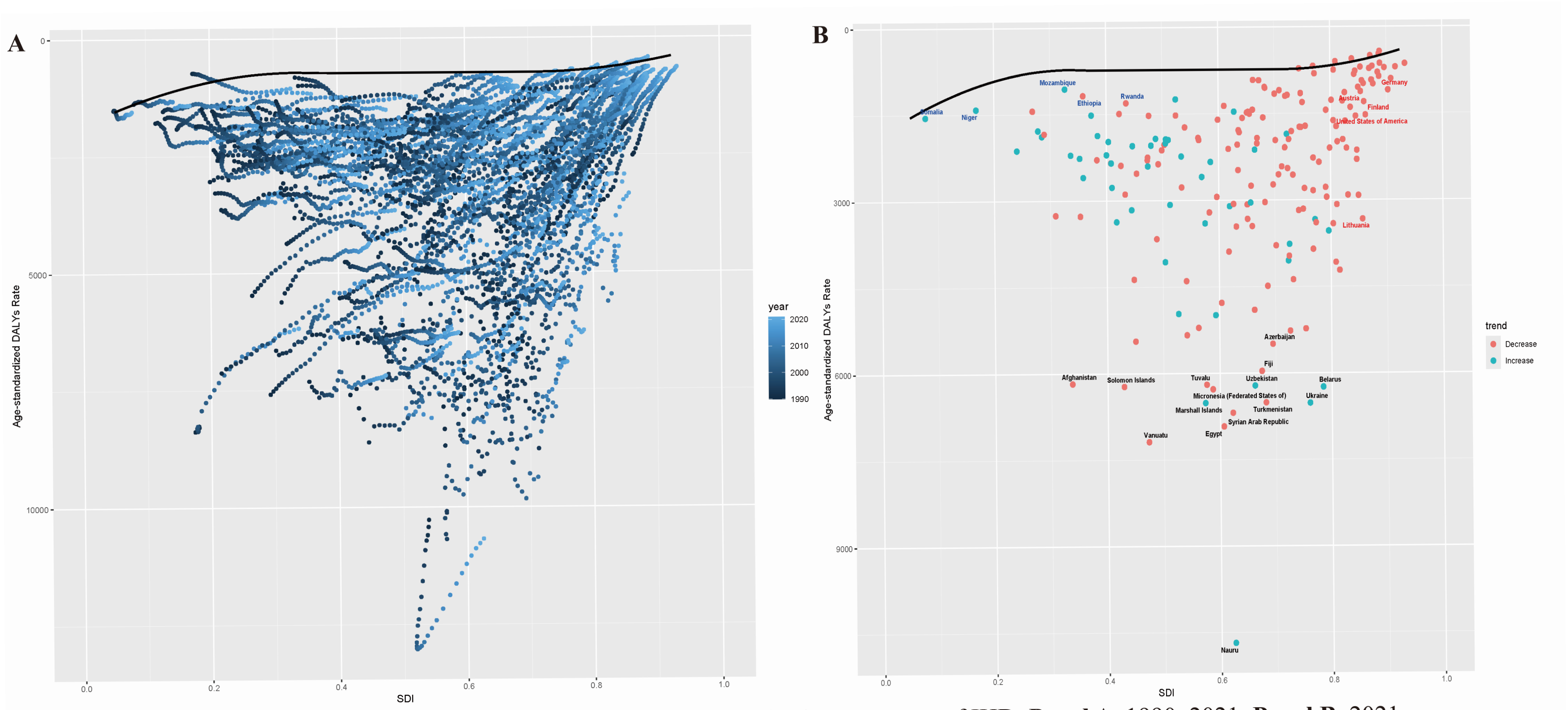

Figure S3. Frontier analysis based on SDI and age-standardised DALYs rate of IHD. **Panel A.** 1990–2021. **Panel B.** 2021. DALY – disability-adjusted life year, IHD – ischaemic heart disease, SDI – sociodemographic index.
